# TRPM8-regulated calcium mobilization plays a critical role in synergistic chemosensitization of Borneol on Doxorubicin

**DOI:** 10.7150/thno.45861

**Published:** 2020-08-13

**Authors:** Haoqiang Lai, Chang Liu, Liyuan Hou, Wenwei Lin, Tianfeng Chen, An Hong

**Affiliations:** 1 Department of Cell Biology & Institute of Biomedicine, National Engineering Research Center of Genetic Medicine, Guangdong Provincial Key Laboratory of Bioengineering Medicine, College of Life Science and Technology, Jinan University, Guangzhou, 510632, China.; 2 Department of Chemistry, Jinan University, Guangzhou, 510632, China.

**Keywords:** Natural Borneol, DOX, TRPM8, Synergism, Calcium mobilization, Non-small cell lung cancer

## Abstract

**Background:** Lung cancer has a high mortality rate and is resistant to multiple chemotherapeutics. Natural Borneol (NB) is a monoterpenoid compound that facilitates the bioavailability of drugs. In this study, we investigated the effects of NB on chemosensitivity in the A549 human lung adenocarcinoma cell line and to elucidate therapeutic molecular target of NB.

**Methods:** The chemosensitivity effects of NB in A549 cells were examined by MTT assay. The mechanism of NB action was evaluated using flow cytometry and Western blotting assays. Surface plasmon resonance (SPR) and LC-MS combined analysis (MS-SPRi) was performed to elucidate the candidate molecular target of NB. The chemosensitizing capacity of NB *in vivo* was assessed in nude mice bearing A549 tumors.

**Results:** NB pretreatment sensitized A549 cells to low doxorubicin (DOX) dosage, leading to a 15.7% to 41.5% increase in apoptosis. This increase was correlated with ERK and AKT inactivation and activation of phospho-p38 MAPK, phospho-JNK, and phosphor-p53. Furthermore, this synergism depends on reactive oxygen species (ROS) generation. MS-SPRi analysis revealed that transient receptor potential melastatin-8 (TRPM8) is the candidate target of NB in potentiating DOX killing potency. Genetically, TRPM8 knock-down significantly suppresses the chemosensitizing effects of NB and inhibits ROS generation through restraining calcium mobilization. Moreover, pretreatment with NB synergistically enhances the anticancer effects of DOX to delay tumor progression *in vivo*.

**Conclusions:** These results suggest that TRPM8 may be a valid therapeutic target in the potential application of NB, and show that NB is a chemosensitizer for lung cancer treatment.

## Introduction

Non-small cell lung cancer (NSCLC) accounts for 75% - 80% of lung cancer, is the leading cause of cancer related mortality worldwide. Despite the tremendous strides that have been made in lung cancer research, the 5-year survival rate for patients with NSCLC remains at 10% - 13%. Chemotherapy remains the therapy of choice for lung cancer, but in NSCLC the response rate of single agents is still lower than 15%. Therefore, it is imperative that new treatments for NSCLC are developed.

Doxorubicin (DOX) is widely used for solid tumor therapy, including in patients with NSCLC, because of its potent cytotoxic properties 1. Studies show that the possible mechanism of DOX towards many malignancies is correlated with targeting topoisomerase II 2, inhibiting anti-apoptosis protein expression 3, and activating p53 4. However, drug-resistance greatly impairs the therapeutic efficiency of DOX, especially for advanced NSCLC treatment. This may result from overexpression of multi-drug resistance proteins 5, NF-κB activation 6, and alteration of topoisomerase II activities. Additionally, dose limiting cardiotoxicity, induction of bone marrow depression induced by higher doses, and poor drug delivery are considered primary DOX therapeutic efficiency obstacles for lung cancer treatment. Combination chemotherapy has been postulated as a promising treatment strategy for NSCLC. Emerging evidence shows the benefit of combination regimens in anticancer therapy, including cisplatin combined with taxanes/gemcitabine 7, and curcumin and 5-fluorouracil 8. However, the search for additional effective agents to improve the efficacy of DOX in cancer therapy remains a topic of intense research.

Natural products have been widely used in traditional medicine, and many phytochemical agents including tripterygium wilfordii 9, betulinic acid 10, and triptolide 11 possess significant anticancer activities. Natural borneol (dextrorotatory borneol ((+)-borneol), NB), a monoterpenoid compound, has been used as an antibacterial, analgesic, and anti-inflammatory agent for 2000 years. The GABAA receptor, TRPA1, and TRPV3 are the direct targets of borneol. The abnormal expression of TRPM channel family proteins is also closely related to the occurrence and development of breast, lung, pancreatic, and prostate cancer 12. TRPM8 overexpression stimulates the progression of prostate cancer, lung cancer, and osteosarcoma 13-15. Therefore, TRPM proteins may be important therapeutic targets of chemotherapy. Accumulating evidence shows that NB can promote the transdermal and mucosal absorption of drugs 16,17. Recently, NB has been shown to enhance the anti-tumor effects of selenocysteine 18, curcumin 19, didemethoxycurcumin 20, cisplatin 21, and paclitaxel 6. Although these studies suggest the potential application of NB in cancer prevention, it is not clear whether NB can be used to improve the therapeutic efficiency of chemotherapy in lung cancer treatment. RNA-Seq 22 and ITRAQ 23 are widely used to explore the targets of small molecules, proteins, and peptides. However, these strategies are still unable to identify direct interactions between protein targets and drug molecules. Surface plasmon resonance (SPR) biosensor technology has been widely used to characterize unmodified biological drugs and identify the macromolecular targets of small molecular agents 24,25. The advantages of this approach are that there is no need for the labeling and real time monitoring of intermolecular interaction. This study was designed to explore whether NB can enhance the inhibitory effect of DOX on the growth of A549 human lung cancer cell line xenograft tumors in nude mice. Combined SPR and LC-MS analysis (MS-SPRi) was used to elucidate the underlying target through which NB serves as chemosensitizer in chemotherapy.

## Materials and Methods

### Chemicals and reagents

Natural borneol (dextrorotatory borneol ((+)-borneol), NB) and synthetic borneol ((±)-borneol and isoborneol) with a purity of > 96% were obtained from the China Institute for the Control of Pharmaceutical and Biological products. Doxorubicin (DOX) (#D1515), 5-Fluorouracil (5-Fu) (#F6627), cisplatin (#BP809), paclitaxel (#T7402), propidium iodide (PI) (#P4864), 2',7'-dichlorofluorescin diacetate (DCFH-DA) (#D6883), rhodamine 123 (Rh 123) (#R8004), bicinchoninic acid (BCA) kit (#BCA1-1KT), Annexin V-FITC apoptosis detection kit (#APOAF-50TST), DAPI (D9542) and 3-(4,5-Dimethylthiazol-2-yl)-2,5-diphenyltetrazolium bromide (MTT) (#M2003) were obtained from Sigma-Aldrich. DMEM and fetal bovine serum (FBS) were obtained from Invitrogen (Carlsbad, CA). Hoechst33342 (#4082) and MitoTracker Green (#9074) were obtained from Cell Signaling Technology (Beverly, MA). Substrates for caspase-3, -8, and -9 were obtained from Calbiochem. Fluo-3 AM was purchased from Eugene, Oregon, USA. Endoplasmic reticulum protein extraction kit (#BB31454) and cell membrane protein extraction kit (#BB3116) were purchased from Bestbio Co. (Shanghai, China). All antibodies used in this study, except for anti-TRPM8 antibody and anti-β-Actin antibody, were purchased from Cell Signaling Technology (Beverly, MA) and detailed information can be found in the supporting information. All chemicals and solvents used were analytically pure.

### Acute toxicity evaluation

There was no information available about the estimated lethal dose and dose series factor of NB in SD rats. Therefore, we used the recommended initial dose of 175 mg/kg and the default dose series factor of 3.2. When using this approach, the dose administered should range from 175, 550, 1750 and 5000 mg/kg, and the dosage should not exceed 5000 mg/kg. One animal was administered 175 mg/kg NB and observed for 48 h. If the first animal survived the initial dose, the next animal received a higher dose. If the animal is dead or dying, the next animal received a lower dose. The manifestation of poisoning and the death of the rats were observed each day after administration of synthetic borneol and NB. The dead rats were dissected and the diseased organs were collected for histopathological examination. Surviving rats were observed continuously for 14 days. The surviving rats were sacrificed by cervical dislocation at the end of the observation period. The LD_50_ and 95% confidence limits were calculated as follows:


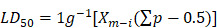
;

S_x50_ = 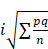;


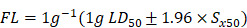


*X*: Logarithm of the maximum dose; *i*: Logarithm of adjacent dose ratios;* p*: Mortality of each dose; *q*: Survival rate of each group of doses; *∑p*: The sum of mortality rates of each group; *n*: Number of animals in each group; *Sx_50_*: Standard deviation of lg *LD_50_*.

### Cell culture and determination of cell viability

A549 human lung adenocarcinoma cells, NCM460 human normal colon epithelial cells, WI-38 human embryonic lung fibroblast cells, H9C2 rat cardiomyoblast cells, normal human hepatic cell line L02, and human normal breast cell line HS578BST were purchased from ATCC (Manassas, VA) and cultured in DMEM supplemented with 10% FBS (FBS was inactivated in water bath at 56 ℃ for 30 min before used) following the suppliers instructions (at 37 ℃, in an atmosphere of 5% CO_2_). This culture condition is also applied for the cell lines experiments. NCI-H460 human lung large cell carcinoma cells were kindly provided by the Stem Cell Bank, Chinese Academy of Sciences. The effects of chemotherapy agents on cell viability were determined by MTT assay. In brief, cancer cells (3 × 10^4^ cells/mL) or normal cells (10 × 10^4^ cells/mL) were plated in 96-well plates and allowed to attach for 24 h at 37 °C in an atmosphere with 5% CO_2_. Different concentrations of agents were added and incubated for the indicated times, then 25 μL of MTT (5 mg/mL in 1 × PBS (10 mM, pH = 7.4)) was added per well and incubated for 5 h. Absorbance at 570 nm was recorded and cell viability was calculated as percentage of untreated groups.

### Clonogenic assay

A549 and NCI-H460 cells (2000/well) were plated in 6-well plates, incubated overnight, then treated with NB (160 μg/mL) for 12 h followed by the indicated DOX concentration. After 24 h, cells were washed with PBS three times, fresh DMEM was added, and cells were allowed to grow for 8 days. After three washes with PBS, cells were fixed with methanol at room temperature for 15 min and stained with 0.5% crystal violet (wt/vol) for 20 min. Images of clonogenic cells were captured using a camera (Life technologies EVOS®FL Auto).

### Cellular DOX uptake

A549 cells (5 × 10^4^ cells/mL) were allowed to attach overnight in 6-well plates. Cells were treated with the indicated concentration of NB for 12 h and then with 250 nM DOX for 0, 1, 2, 4, and 8 h. Cells were stained with DAPI for another 10 min. Intracellular DOX accumulation was examined by microscopy (Life technologies EVOS®FL Auto). After trypsinization, cells were collected by centrifugation (350 g, 5 min) and suspended in 300 μL PBS. Intracellular DOX accumulation was evaluated using Flow cytometry assays (Beckman, CytoFLEX S). Data were analyzed by Flowjo_V10 software.

### Evaluation of P-glycoprotein (P-gp) function

A549 cells (8 × 10^4^ cells/mL) were plated in a 96 well plate overnight. NB (40, 80, and 160 μg/mL) was added and cells were incubated for 12 h. Another group of cells were treated with 160 μg/mL NB for 0, 3, 6, 9, and 12 h. Rhodamine 123 (5 μM) was added and incubated for 1 h at 37 °C. After three washes with PBS to remove extracellular rhodamine 123, cells were lysed in Triton X-100 solution (1% in 1 × PBS (10 mM, pH = 7.4)) for 30 min. rhodamine 123 fluorescence was recorded using a fluorescence microplate reader with excitation and emission wavelengths of 488 nm and 530 nm, respectively.

### Flow cytometric analysis

Annexin-V and PI staining assays were used to evaluate the effects of combined NB and DOX treatments on apoptosis. In brief, A549 cells (3 × 10^4^ cells/mL) were plated in a 6 cm dish and incubated overnight. Then, cells were treated with 160 μg/mL NB for 12 h followed by 250 nM DOX for 72 h. After trypsinization, cells were collected by centrifugation at 350 g for 5 min and washed once with cold PBS. Cells were resuspended in 300 μL 1 × Binding Buffer and 5 μL of Annexin V-FITC was added and incubated in the dark for 15 min. After that, 5 μL of PI was added and incubated for 5 min. Finally, 200 μL 1 × binding buffer was added and apoptotic cells were analyzed by flow cytometry (CytoFLEX S, Beckman).

For analysis of cell cycle distribution, A549 cells (3 × 10^4^ cells/mL) were plated in a 6 cm dish and incubated overnight. Cells were treated with different concentrations of NB and DOX for the indicated times. Cells were then collected and fixed overnight with 70% cold ethanol. Cells were collected by centrifugation and stained with PI for 30 min in the dark or stained with Hoechst 33342 for 30 min. Flow cytometry was used to analyze the cell cycle distribution and ModFit LT5.0 software was used to analyze the results.

### Evaluation of mitochondrial structure and mitochondrial electric potential (ΔΨ)

A549 cells (3 × 10^4^ cells/mL) were plated in a 2 cm glass dish overnight. MitoTracker Green CMXRos and Hoechst 33342 were used to stain the structure of mitochondria and nucleus, respectively. Cells were treated with 160 μg/mL NB for 12 h followed by 250 nM DOX for 0, 1, 2, 4, or 8 h. Mitochondrial structure was examined under a fluorescent microscope (Life technologies EVOS®FL Auto, 100 ×). Mitochondria electric potential (*Δψ*) examination was performed as previously described 26. Fluorescence intensity was recorded using a Biotek Microplate System with excitation and emission wavelengths of 550 and 600 nm, respectively, for red fluorescence, and 485 and 535 nm, respectively for green fluorescence.

### Examination of ROS generation

The DCFH-DA fluorescence assay was used to examine intracellular ROS accumulation 27. In brief, cells were stained with the DCFH-DA (10 μM) fluorescence probe for 30 min at 37 °C in the dark. Cells were washed twice with PBS to remove the unlabeled probe. Then 10^6^ cells per well were incubated with 160 μg/mL NB and 250 nM DOX for 2 h, and changes in fluorescence intensity were recorded using a Biotek Microplate System with excitation wavelength and emission wavelengths of 488 and 525 nm, respectively. Relative fluorescence intensity of treated cells is expressed as percentage of control groups (as 100%).

### Caspase activity assay

A549 cells (10 × 10^4^ cells/mL) were plated in 10 cm dishes for 24 h. Cells were then treated with 160 μg/mL NB for 12 h followed by 250 nM DOX for 72 h. Total cell lysate was extracted and the concentration of total cell protein was examined by BCA assay. Protein (100 μg/well) was incubated with 5 μL caspase substrates at 37 °C for 2 h in the dark. Fluorescence intensity was recorded using a Biotek Microplate System and excitation and emission wavelengths of 380 and 440 nm, respectively.

### Screening and identification of NB-captured proteins in A549 cells

MS-SPRi was used to elucidate the protein target of NB in A549 cells. In brief, total A549 cell proteins were extracted by incubating cells in lysis buffer, and proteins were passed through the nanosensor chip (Betterways Inc., China) to identify those immobilized by NB. The proteins or peptides captured by NB were digested *in situ* by trypsin, and identified using HPLC-MS/MS. MaxQuant software (COX LAB, version 1.3.0.5) used to analyze MS data. The captured peptides and proteins were analyzed through using Proteome Discoverer (Thermo Fisher Scientific, version 1.7) by retrieval in UniProtKB/Swiss-Prot.

### siRNA transfection

Two kinds of siRNA were used to knockdown TRPM8 transmembrane and non-transmembrane regions. The siRNA duplexes were obtained from Sangon Biotech Co., Ltd. (Shanghai, China) and the siRNA sequences targeting the transmembrane region were: siTRPM8, sense (5'-3') GGATGCTGATCGATGTGTT and antisense (5'-3') AACACAUCGAUCAGCAUCCTT. Another siRNA (siTRPM8-1) targeted the region upstream of the transmembrane region with the targeted sequence: ACGCGGAAATCCTTTACGA. The siRNA targeting the androgen receptor (AR) was: sense (5'-3') GAGCACTGAAGATACTGCTGA and antisense (5'-3') ACGUGACACGUUCGGAGAATT. A549 cells (5 × 10^4^ cells/mL) were plated in 96 well plates, or 10 × 10^4^ A549 cells/mL were plated in 6 well plates, for 24 h and siRNA (50 nM and 100 nM siRNA was used for 96 well plates and 6 cm dishes, respectively) transfection was conducted as per the manufacturer's instructions (Invitrogen). After transfection for 48 h, cells were pretreated with 160 μg/mL NB for 12 h followed by 250 nM DOX for 48 h. The siRNA control was generated using the same process. Cell viability was evaluated by using the MTT assay and knockdown efficiency was evaluated by Western blotting.

### Evaluation of intracellular Ca^2+^ mobilization

Assessment of NB-induced intracellular Ca^2+^ mobilization in A549 cells was carried out using flow cytometry. In brief, A549 cells were harvested by centrifugation at 350 g for 5 min. Then cells (10^5^ cells/mL) were suspended in 2 mL 10 mM HEPES solution (without calcium but containing 0.05% Pluronics F127) and stained with 5 μM Fluo-3 acetomethyl ester (Eugene, Oregon, USA) for 20 min at 37 °C in the dark. HEPES solution (10 mL with 1% FBS, but without calcium) was added and incubated for another 40 min at 37 °C in the dark. Cells were then harvested and washed twice with HEPES (containing 0.099% sucrose and 0.35% BSA). Labeled cells were suspended in 500 μL HEPES solution (without calcium) and collected for 20 s to acquire baseline fluo-3 fluorescence. Then, indicated concentrations of NB or menthol (500 μM) were quickly added and the changes in green fluorescence were recorded by setting up the stop-time condition at 120-200s.

### Immunofluorescence

A549 cells were plated in 2 cm glass bottom dishes at a density of 4 × 10^4^ cells/mL and incubated overnight. Cells were fixed with 4% formalin in PBS for 20 min followed by two washes in PBS. Then cells were blocked in 5% BSA for 1 h and incubated with primary anti-TRPM8 antibody (1:200, allomone, ACC-049) overnight at 4 °C. After three washes with PBS, the secondary antibody (Alexa Fluor® 488, 1:500) was added and incubated for 1 h at room temperature. Finally, TRPM8 expression of was analyzed using a fluorescence microscope (Life technologies EVOS®FL Auto, 100 ×).

### Western blot analysis

A549 cells (10 × 10^4^ cells/mL) were plated in a 10 cm dish for 24 h. Cells were treated with 160 μg/mL NB for 12 h and then incubated with 250 nM DOX for 72 h. Cells were lysed with lysis buffer (150 mM NaCl, 1% NP-40, 0.1% SDS, 2 μg/mL aprotinin, 2 μg/mL leupeptin, 1 mM PMSF, 1.5 mM EDTA, and 1 mM NaVanadate) and total cell protein was collected by centrifugation at 12040 g for 15 min. The BCA assay was used to adjust the extracted protein concentration. Protein (40 μg) was separated by 10% SDS-PAGE electrophoresis. Proteins were then transferred to nitrocellulose membranes, blocked with 5% BSA blocking solution, and incubated with primary antibodies (1:5000) at 4 °C overnight. Membranes were then incubated with second antibody (1:2000). Protein expression was visualized using a Tanon 5200 chemiluminescence imaging system after incubation with HRP Substrate (Sigma). Protein bands were quantified using Quantity one software (Bio-Rad).

### *In vivo* anti-tumor activity

All animal experiments were performed in accordance with the Guidelines for Care and Use of Institutional Animal Care and Use Committee of Jinan University Laboratory Animal Center and approved by the Animal Ethics Committee of Jinan University. About 1 × 10^6^ A549 cells in PBS were subcutaneously injected into the right oxter of nude mice. After the tumor volume reached 50-60 mm^3^, mice were randomly divided into six groups (8 mice/group): saline; intravenous administration (*i.v.*) DOX; *i.v.*NB; oral administration (*p.o.*)NB; *i.v.*NB + *i.v.*DOX; and *p.o.*NB + *i.v.*DOX groups. The intravenous dosage of NB (0.002 g/kg), oral dosage of NB (0.2 g/kg), and intravenous dosage of DOX (0.004 g/kg) were selected as previously described 28,29. Mice were euthanized and the tumor tissues were harvested, photographed, and weighed at the end of the experiments. Meanwhile, serum from the venous blood of all groups was harvested and used for serum clinical chemistry analysis.

### DOX extraction from tumor tissues

DOX from was extracted tumor tissues as previously described 30. Tissues (0.2 g) in 1 mL ultrapure water were subjected to ultrasonication. Then, 200 μL of 33% AgNO_3_ (w/v in H_2_O_2_) was added and vibrated for 10 min. Cold isopentanol (4 mL) was added and vibrated for another 10 min. After centrifugation (2080 g, 10 min), the supernatant was collected and the fluorescence intensity was recorded using examination and excitation wavelengths of 470 nm and 595 nm, respectively. Meanwhile, a standard curve was produced using DOX concentrations that varied from 0.025, 0.05, 0.5, 1.0, 5.0, to 10 μg/mL.

### Statistical analysis

Data are expressed as mean ± SD. Statistical analysis was performed using One-Way ANOVA in SPSS statistics 25 (SPSS statistics 25; SPSS, Inc. Chicago, IL). * and ** are denoted as *P* < 0.05 and *P* < 0.01, respectively.

## Results

### NB potentiates cytotoxicity of DOX against A549 cells through enhancing cellular uptake

**Figure 1:**
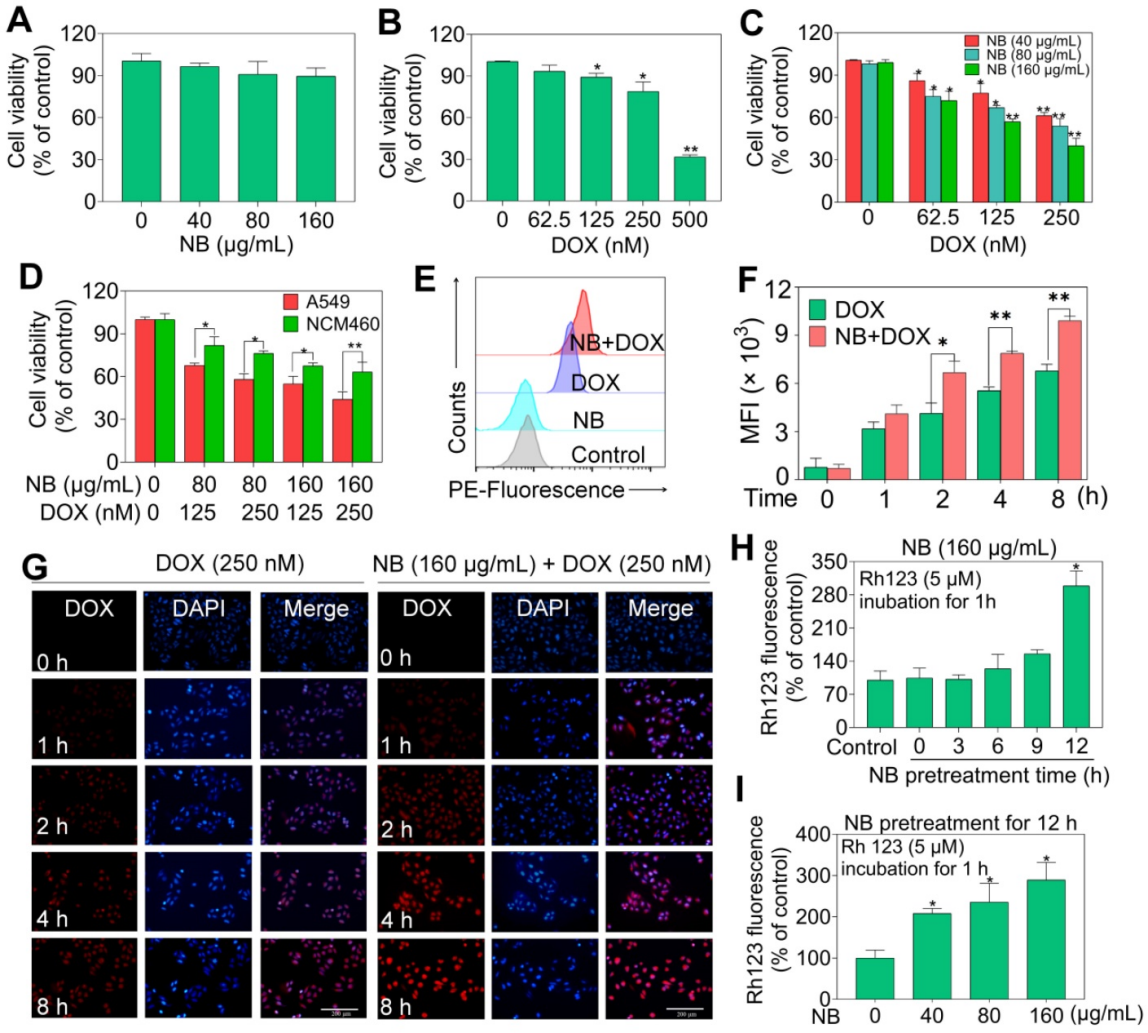
** NB augments DOX cell growth inhibitory effects in A549 cells through enhancing cellular DOX uptake.** Effects of different concentrations of NB (**A**) and DOX (**B**) on cell growth in A549 cells. **(C)** NB enhances DOX-mediated killing in A549 cells in a dose-dependent manner. (**D**) Growth inhibition induced by combination treatment with NB (160 µg/mL) and DOX (250 nM) in NCM-460 cells. (**E**) Flow cytometric histogram of DOX cellular accumulation. (**F**) Mean red fluorescence intensity of DOX in A549 cells. (**G**) Representative fluorescence images of the intracellular DOX uptake in A549 cells. Evaluation of P-gp function after cells treated with NB (160 µg/mL) for different times (**H**) or cells treated with different doses of NB for 12 h (**I**). A549 cells were pretreated with NB (160 µg/mL) for 3, 6, 9 and 12 h or cells preincubated with NB (40, 80 and 160 µg/mL) for 12 h and then co-treated with Rh 123 (5 µM) for another 1 h. *P < 0.05, **P < 0.01, n = 3.

The acute toxicity of dextrorotatory borneol ((+)-borneol, NB) and synthetic borneol (a mixture of (±)-borneol and isoborneol) was examined. The median lethal dose (LD_50_) of synthetic borneol was 3129 mg/kg with a 95% confidence interval of 1750~5000 mg/kg. The LD_50_ of NB was 5000 mg/kg with a 95% confidence interval of 2016~9810 mg/kg (**Table S1**). Moreover, significant pathological changes were observed in the liver, spleen, and lung of SD rat treated with 5000 mg/kg of synthetic borneol. Slight inflammatory effects were observed in the stomach and intestinal mucosa in rat treated with synthetic borneol and NB (5000 mg/kg) (**Figure S1**). These results suggest that NB has a higher safety index than synthetic borneol, and NB was selected for the chemo-sensitizing study. As illustrated in**Table S2**, NB enhanced the cytotoxic effects of DOX against A549 cells and IC_50_ value of DOX decreased to 180 ± 0.06 nM). Similar enhancement effects of NB on 5-FU, paclitaxel, and cisplatin were also observed. These results suggest that NB has the capacity to sensitize A549 cells to chemotherapeutic agents, and we selected NB and DOX for further study. NB exhibits slight cell growth suppression effects in A549 cells (**Figure 1A**). DOX treatment alone slightly represses A549 cell growth at a concentration range of 60 nM to 250 nM (**Figure 1B**). However, obvious cytotoxicity effects were observed when cells pretreated with NB for 12 h followed by low dose DOX for 72 h with the suppression ratio increasing up to 40.26%. Long term clonogenic assay results were consistent with these results (**Figure S2A**-**B**). Similar enhanced effects induced by combination treatment with NB and DOX were also observed in NCI-H460 cells (**Figure S3**). Furthermore, we explored the cytotoxicity of NB and DOX in normal colonic NCM-460 cells and found that NCM-460 cells were less inhibited than were A549 cells (**Figure 1D**). Additionally, we found that NB pretreatment did not augment the cytotoxicity effects of DOX against several normal cell lines including WI-38, L02, H9C2, and HS578BST cells (**Figure S4**). Together, these results suggest that NB potentiates the effect of DOX to induce cytotoxicity effects mainly in tumor cells.

To investigate the underlying mechanism of the cytotoxicity effects induced by NB and DOX, intracellular DOX accumulation was examined. NB pretreatment dramatically augments the intracellular DOX accumulation compared to DOX treatment alone. Intracellular accumulation of DOX in A549 cells examined by flow cytometry assay also confirmed these results, as indicated by the flow histogram (**Figure 1E**) and the increased red fluorescence intensity in the combination treatment groups of NB and DOX when compared to DOX alone treatment groups (**Figure 1F**) These results are further confirmed by the images of fluorescence in cells (**Figure 1G**). Moreover, P-glycoprotein (P-gp) mediated drug resistance is one of the major causes of DOX-resistance in NSCLC 31. P-gp functional analysis showed that treatment with 160 μg/mL NB for 12 h significantly enhanced the accumulation of Rhodamine 123 in A549 cells (**Figure 1H**). Additionally, increased cellular red Rhodamine 123 fluorescence was observed in cells treated with 40, 80, and 160 μg/mL NB for 12 h (**Figure 1I**). Together, these results suggest that NB inhibits P-gp function and potentiates the cytotoxic effects of DOX in A549 cells.

### NB synergizes with DOX to induce apoptosis through activating the caspase cascade in A549 cells

**Figure 2:**
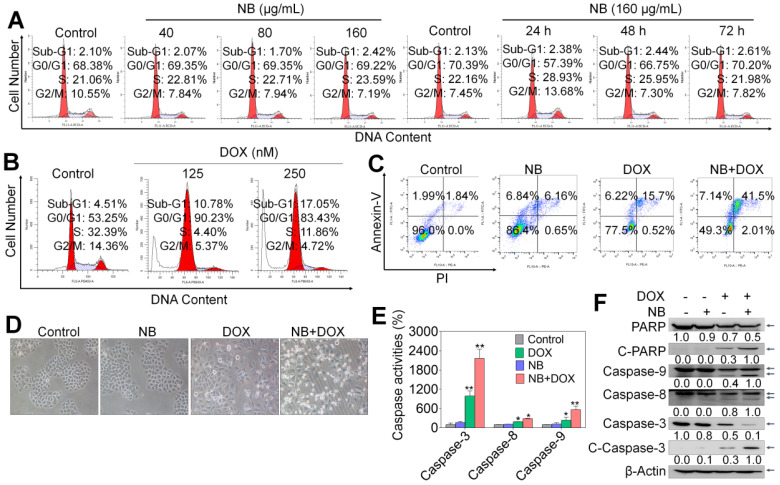
** NB potentiates DOX-induced apoptosis in A549 cells.** Effects of NB (**A**) and DOX (**B**) on the cell cycle distribution of A549 cells. (**C**) Pretreatment with NB (160 µg/mL) enhances 250 nM DOX-induced apoptosis in A549 cells. (**D**) Representative images of A549 cells after treatment with NB (160 µg/mL) and DOX (250 nM) for 72 h. (**E**) Activation of caspase-3, -8, and -9 induced by combination treatment with NB (160 µg/mL) and DOX (250 nM). In brief, 100 µg of protein was incubated with 5 µL of specific fluorescent substrates for caspase-3, -8, and -9 for 2 h and the fluorescence intensity was measured using a Biotek Microplate System. (**F**) Western blotting assay of caspase-3/-8/-9, PARP, cleaved-PARP, and cleaved-caspase-3 protein expression levels. *P < 0.05, **P < 0.01, n = 3.

We used flow cytometry to assess the mode of cell death triggered by combination NB and DOX treatment. No significant cell cycle distribution changes were observed in NB treatment groups, while significant accumulated populations of Sub-G1 (17.05%) and G0/G1 phase cells were observed after DOX incubation (**Figure 2A**-**B** and**Figure S5**). These results suggest that DOX can trigger cell cycle arrest and apoptosis in A549 cells. However, significant apoptotic effects were observed in cells pretreated with 160 μg/mL NB and then co-treated with 250 nM DOX, as illustrated by the increased population of Annexin V and PI double positive cells (**Figure 2C**). Apoptotic cells accounted for 13.0% of cells in NB treatment groups and 21.92% of cells in DOX incubation groups. However, pretreatment with 160 μg/mL NB significantly enhanced the killing potency of DOX with the proportion of apoptotic cells increasing to 41.5%. This was confirmed by morphological changes in treated cells (**Figure 2D**). These results indicate that NB sensitizes A549 cells to DOX-mediated killing through inducing apoptosis.

Caspase-8 and caspase-9 activation are confirmed to be a major mediator of activation of extrinsic and intrinsic apoptosis pathway, respectively. To confirm the apoptosis inducing effects triggered by the combined treatment with NB and DOX, a fluorometric assay was used to examine caspases activation. Both NB and DOX were able to induce slight activation of caspase-3, -8, and -9 (**Figure 2E**). However, cells pretreated with 160 μg/mL NB and then incubated with 250 nM DOX demonstrated a synergistic increase in caspase-3, -8, and -9 activation, indicating that extrinsic and intrinsic apoptosis pathways were activated. Western blotting assay results showed significant cleavage of caspase-9, -8, -3, and PARP and further strengthen these findings (**Figure 2F** and**Figure S6**). Together, these results demonstrate that NB enhances DOX-induced A549 cell death by inducing apoptosis through activating extrinsic and intrinsic apoptotic pathways.

### ROS-mediated pathway activation contributes to the apoptotic effects induced by NB and DOX

**Figure 3:**
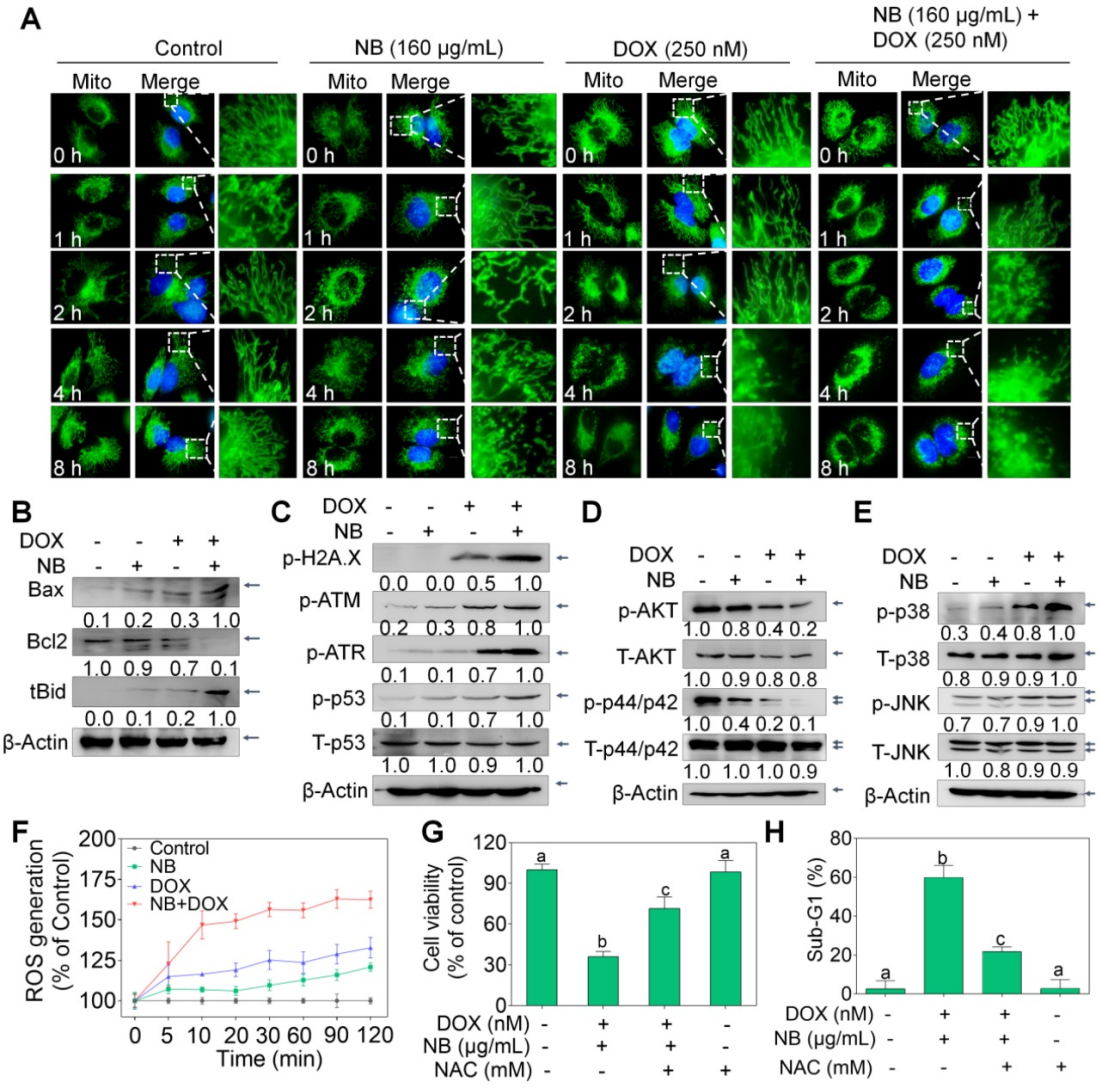
** NB and DOX synergize to activate ROS-mediated pathways in A549 cells.** (**A**) Alteration of mitochondrial structure induced by the combined treatment with NB (160 µg/mL) and DOX (250 nM) in A549 cells (magnification, 100 ×). (**B**) Bcl-2, Bax, and truncated Bid protein expression in A549 cells after incubation with NB and DOX. (**C**) NB (160 µg/mL) potentiates 250 nM DOX-induced phosphorylation of ATM, ATR, p53, and histone. Combination treatment with NB and DOX suppresses phospho-AKT and -ERK expression (**D**), while upregulating phospho-p38MAPK and phospho-JNK expression levels (**E**). (**F**) NB (160 µg/mL) enhances 250 nM DOX-induced ROS generation in A549 cells. (**G**) NAC pretreatment elevates cell viability induced by combination treatment with NB (160 µg/mL) and DOX (250 nM). (**H**) NAC reduces the apoptosis inducing capacity of NB (160 µg/mL) and DOX (250 nM). Cells were treated with 5 mM NAC for 2 h prior to incubation with NB (160 µg/mL) and DOX (250 nM). Three independent experiments were performed for this assay. Bars with different characters (a-c) indicate statistical difference at *P* < 0.05, (n = 3).

Extrinsic and intrinsic apoptosis signals converge in the mitochondria. Therefore, the MitoTracker Green probe was used to examine mitochondrial integrity. Compared to the extensively interconnected and filamentous network of mitochondria in the control treatment groups, treatment with 160 μg/mL NB triggered some mitochondrial structural damage after 8 h incubation. Slightly destroyed mitochondrial structure can also be observed in cells treated with 250 nM DOX alone for 4 h. However, mitochondrial fragmentation was observed at 2 h after incubation with 160 μg/mL NB and 250 nM DOX, notable damage was observed at 4 h, and this became progressively worse at 8 h (**Figure 3A**). Mitochondrial membrane potential (∆*ψ*m) depletion confirmed that the mitochondria were damaged (**Figure S7**). Furthermore, we found that Bcl-2 expression was strongly inhibited by the combined treatment with 160 μg/mL NB and 250 nM DOX, while tBid and Bax expression was significantly upregulated (**Figure 3B** and**Figure S8**). These results suggest that NB enhances DOX killing potency and is highly correlated with mitochondria dysfunction.

Triggering DNA damage is a major mechanism of DOX action 32, and may induce mitochondria-mediated apoptosis through p53 activating. We found that 160 μg/mL NB significantly enhanced 250 nM DOX-induced DNA damage, as evidenced by upregulated levels of phosphorylated ATM, ATR, and histone (Ser 139), and of phosphorylated p53 (Ser 15) (**Figure 3C** and**Figure S9A**). These results indicate that DNA damage-mediated p53 activation contributes to the sensitizing effects of NB on DOX in A549 cells.

Mitogen-activated protein kinase (MAPK) and phosphoinositide 3-kinase (PI3K)/AKT pathways play important roles in the proliferation and metastasis of cancer cells. We evaluated whether the NB and DOX combination treatment could affect the function of these kinases. Combined treatment with 160 μg/mL NB and 250 nM DOX dramatically suppressed phosphorylated-AKT and phosphorylated-ERK and upregulated the phosphorylation of p38 MAPK (Thr180/Tyr182) and SAPK/JNK (Thr183/Tyr185) (**Figure 3D**-**E and Figure S9B-C**). These results suggest that NB synergizes with DOX to induce A549 cell apoptosis through AKT and ERK inhibition while stimulating the activation of p38 MAPK and JNK.

Evidence has revealed the pivotal roles of ROS in the anticancer mechanisms of DOX 33. Moreover, ROS plays a regulatory role in the modulation of MAPK and PI3K/AKT pathways 33. Both 160 μg/mL NB and 250 nM DOX triggered ROS generation, and strong upregulation of ROS accumulation was achieved when cells were incubated with the combined treatment of NB and DOX (**Figure 3F**). Moreover, pretreatment with N-acetyl-L-cysteine (NAC), a thiol reducing antioxidant, significantly inhibited the cell growth suppression and apoptosis induced by combined treatment with NB and DOX (**Figure 3G**-**H**), suggesting the upstream role of ROS. Taken together, these results demonstrate that NB dramatically augments DOX-triggered apoptosis and requires the activation of the ROS-mediated DNA pathway.

### NB may enhance the chemosensitivity of A549 cells to DOX through targeting TRPM8

**Figure 4:**
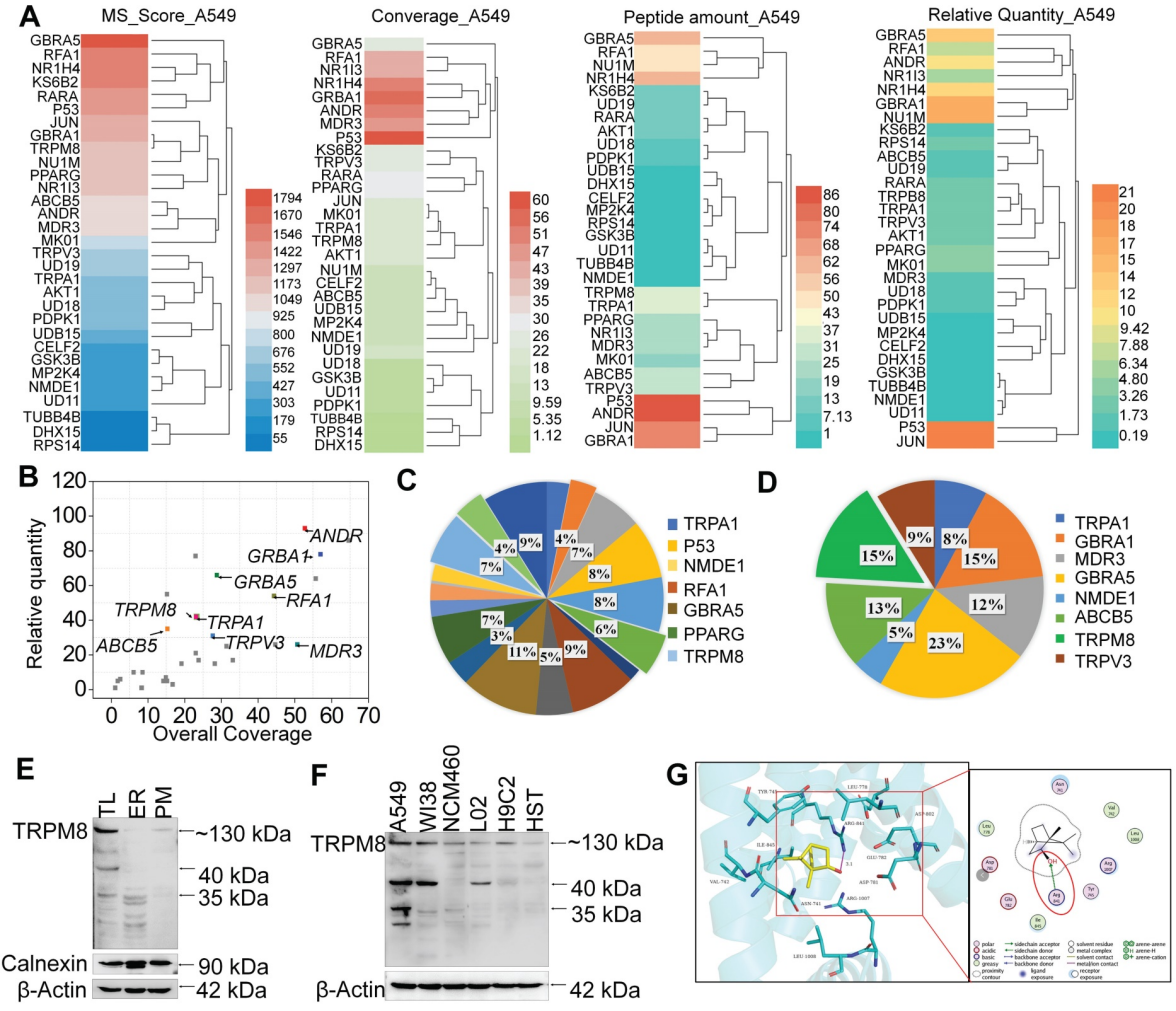
** TRPM8 is the candidate target of NB.** (**A**) MS score and relative quantity of captured proteins by NB. (**B**) Captured proteins are displayed in scatterplots. Go analysis of the captured targets in response to stress (**C**) and transmembrane transporter activity (**D**). (**E**) TRPM8 protein expression in A549 cells. TL (Total cell lysate), ER (endoplasmic reticulum fractions), PM (plasma membrane fractions). (**F**) Differences in TRPM8 protein expression levels in A549 cells and normal cell lines. HST (HS578BST cells). (**G**) Docking conformation of NB and TRPM8 by using Molecular Operating Environment. The PDB ID of TRPM8 is 6BPQ. Interactions between NB and the key residues of TRPM8 in 3D (left panel) and 2D (right panel) models are shown. H-bond interactions between NB and the active site residues (Arg^841^) of TRPM8 are indicated by the red circle.

SPR biosensor technology is widely used for peptide and small molecule drugs screening 34. Since NB enhances the anticancer activities of DOX, MS-SPRi technology was exploited to elucidate the candidate targets of NB. The MS score is used to evaluate the binding strength of NB on proteins, and Peptide Spectrum Matches (PSMs) represents the number of peptide map matches. As shown in **Figure 4A**-**B,** 31 A549 cell lysis proteins were captured by NB. Gene Ontology cluster analysis in Biological Process revealed that most of the captured proteins are involved in cellular process regulation. Additionally, 19 of the 31 proteins take part in the regulation of stress response. Moreover, 21% of these 19 proteins are ion channel proteins, including GABRA5, TRPM8, TRPA1, and TRPV3 (**Figure 4C-D**). The MS score of GABRA5 (PSMs = 66) was the highest followed by TRPM8 (PSMs = 31). Since GABRA5 mainly participates in neuron-neuron synaptic transmission regulation 42, we speculated that TRPM8 may be an important target of NB, and TRPM8 was selected for further study.

**Figure 5:**
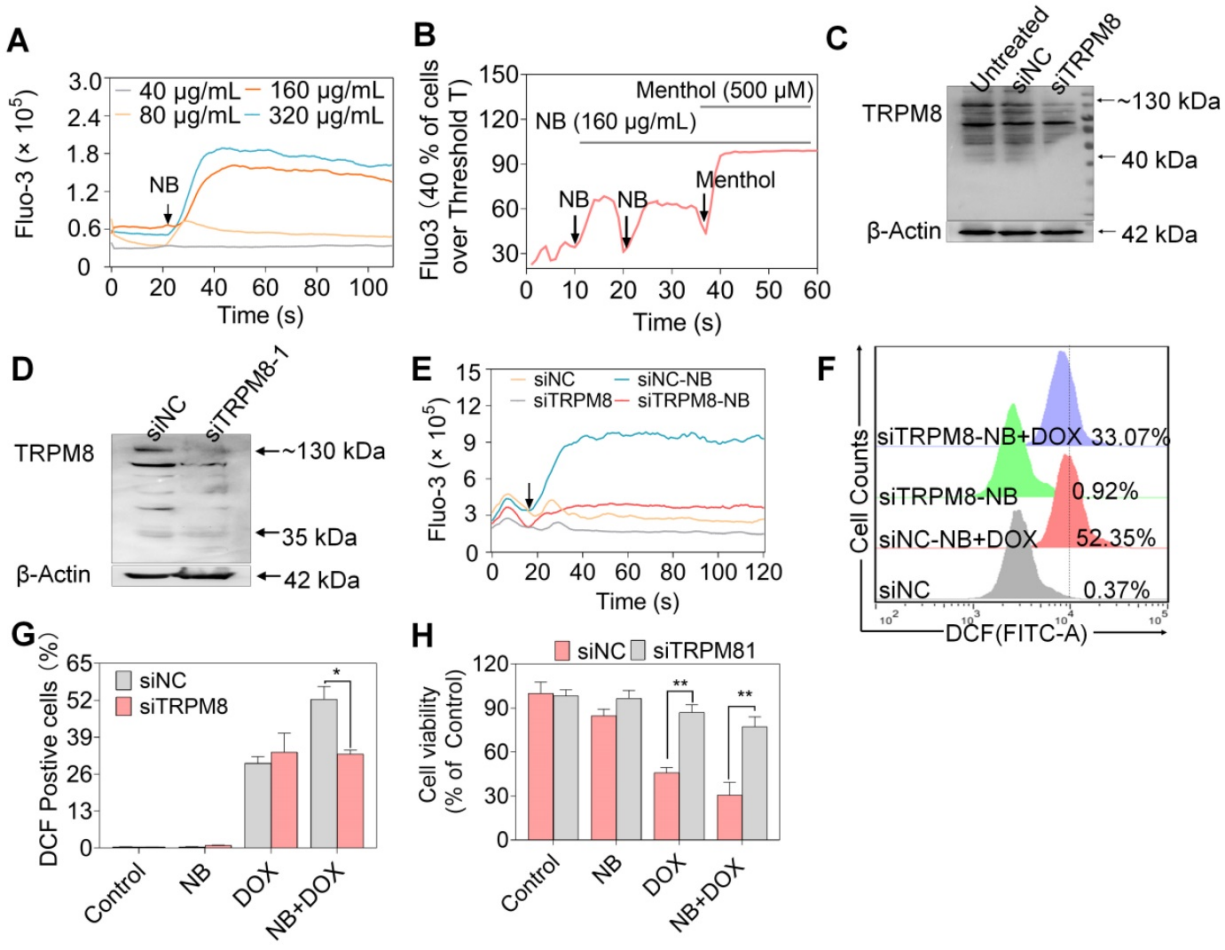
** The synergy between NB and DOX to inhibit A549 cell growth involves TRPM8-meditated Ca^2+^ mobilization.** (**A**) NB evokes Ca^2+^ concentration changes in the cytoplasm in a dose-dependent manner. (**B**) Ca^2+^ changes in the cytoplasm in the presence of NB (160 µg/mL) and menthol (500 µM). (**C**) TRPM8 protein expression levels in A549 cells after transfection with siRNA (100 nM in 6 well plate) targeting the transmembrane of TRPM8. (**D**) TRPM8 expression levels in cells transfected with siRNA (100 nM in 6 well plate) targeting the region upstream of the transmembrane region. The target TRPM8 gene sequence is ACGCGGAAATCCTTTACGA. (**E**) Intracellular Ca^2+^ accumulation triggered by NB (160 µg/mL) in TRPM8 knockdown cells. (**F**) Histogram of intracellular ROS accumulation in TRPM8 knockdown cells. (**G**) The DCF positive cell population in TRPM8 knockdown cells after treatment with NB (160 µ/mL) and DOX (250 nM). (**H**) Cell viability in TRPM8 knockdown A549 cells after treatment with NB (160 µ/mL) and DOX (250 nM) after transfection with siTRPM8-1 (50 nM). *P < 0.05, **P < 0.01, n = 3. Arrow indicates the addition of the indicated concentration of NB or menthol to A549 cells.

TRPM8 is a lung cancer marker 35. TRPM8 is overexpressed in lung adenocarcinoma, and TRPM8 expression is negatively correlated with patient survival according to the TCGA and PROGgene databases (**Figure S10**) 36,37. We next employed immunocytochemistry and Western blotting to evaluate the TRPM8 expression in A549 cells. Obvious green fluorescence was observed in A549 cells, suggestive of TRPM8 expression in A549 cells (**Figure S11**). To further confirm these results, TRPM8 expression in total membrane protein extracts and endoplasmic reticulum protein extracts were examined. TRPM8 is expressed in the plasma membrane and endoplasmic reticulum (**Figure 4E**). This is supported by the presence of full length TRPM8 in plasma membrane extracts and of the small length of TRPM8 in endoplasmic reticulum extracts. Furthermore, higher expression level of TRPM8 was observed in A549 cells than in normal cells (**Figure 4F**). To investigate whether there is direct interaction between NB and TRPM8, Molecular Operating Environment software was used to determine the docking conformations of NB and TRPM8 (**Figure 4G**). NB was able to form a hydrogen bond with the TRPM8 Arg^841^ side chain (the red circle in **Figure 4G**), which is the critical residue for menthol activation of human TRPM8 38. Additionally, the hydrophobic bridge ring of NB can also form hydrophobic interactions with hydrophobic amino acids Tyr^745^, Ile^845^, and Val^742^. Tyr^745^ is the critical residue for menthol binding, and displays central roles in ligand-dependent gating in TRPM8 39. These results suggest that NB may activate TRPM8 through direct interaction. TRPM8 is an important calcium channel protein 40. Therefore, we assessed whether NB could affect the intracellular dissociative calcium concentration in A549 cells. The intracellular fluorescence signal of A549 cells was enhanced after NB addition in a concentration dependent manner, indicating the capacity of NB in inducing calcium mobilization (**Figure 5A**). Additionally, we examined calcium mobilization after the addition of the TRPM8 agonist, menthol, and found that menthol could induce Ca^2+^ efflux in A549 cells. Increased Fluo-3 fluorescence was observed after the addition of NB (160 μg/mL), and this fluorescence intensity was enhanced after the addition of menthol (500 μM), which suggests that menthol can directly bind and activate TRPM8 in A549 cells (**Figure 5B**). These results confirm that NB can trigger calcium mobilization in A549 cells. To investigate whether NB affects calcium accumulation through interacting with TRPM8, short siRNAs targeting the TRPM8 transmembrane region and upstream of the transmembrane region were introduced. As shown in** Figure 5C**, the expression levels of full length TRPM8 protein (about 130 kDa) and of the canonical TRPM8 isoform (about 40 kDa) were obviously inhibited after 48 h siRNA transfection. In cells transfected with siRNA (siTRPM8-1) targeting the upstream of the transmembrane region, full length of TRPM8 expression was greatly inhibited and expression of the shorter TRPM8 isoform was less inhibited (**Figure 5D**). The intracellular calcium signal induced by NB was significantly downregulated in TRPM8 knockdown cells when compared with siNC control groups (**Figure 5E**). These results indicate the important role of TRPM8 in calcium immobilization triggered by NB stimulation. We also found that TRPM8 knockdown reduced the accumulation of intracellular ROS, as indicated by the overlay flow histogram. The population of DCF positive cells decreased from 52.35% to 33.07% (**Figure 5F-G**) after combined treatment with NB and DOX. To evaluate the silencing effects on the anticancer activities of NB and DOX after TRPM8 knockdown, siTRPM8-1 was selected and the MTT assay was employed. We found that the sensitizing effects of NB on DOX in A549 cells were dramatically inhibited after TRPM8 knockdown (**Figure 5H**). In addition to TRPM8, we found that the MS score of the AR was 1077.15 and of the PSMs was 93, indicating that AR may also participate in the sensitizing effects of NB. Furthermore, patients with high AR expression levels have a lower survival rate 37. To evaluate whether the synergistic effects of NB and DOX required interaction between NB and AR, AR knockdown assays were performed. No significant changes in anticancer effects triggered by NB and DOX were observed between siAR knockdown groups and siNC control groups (**Figure S12**). This suggests that AR may not participate in the regulation of NB and DOX anticancer synergism. Taken together, these results suggest that NB may synergize with DOX to achieve potent anticancer efficiency by triggering intracellular Ca^2+^ mobilization through interacting with TRPM8.

### NB augments the anti-tumor activities of DOX *in vivo*

**Figure 6:**
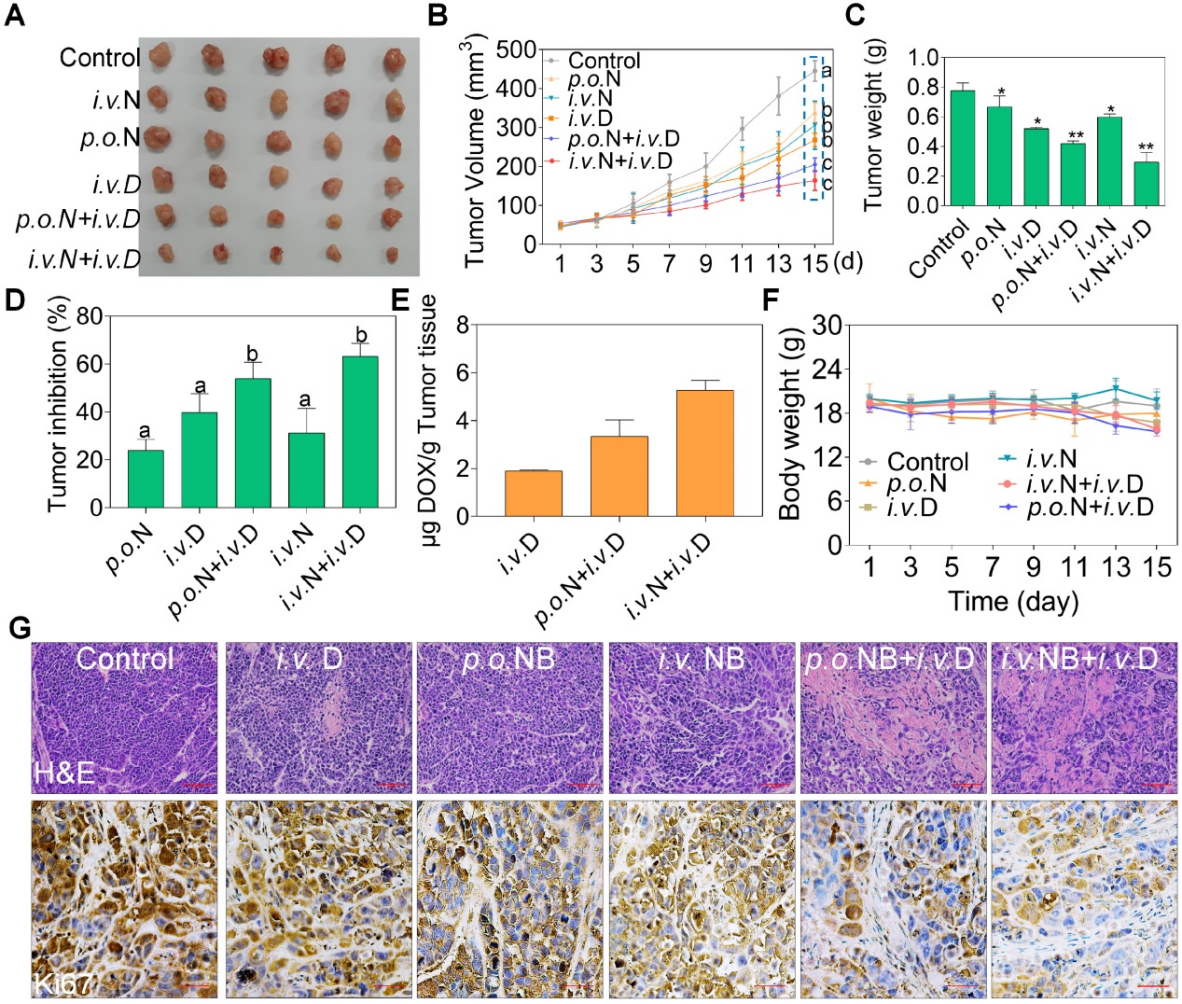
** NB potentiates DOX anti-tumor activities *in vivo*.** Representative photograph of tumors in different treatment groups (**A**). Tumor volume (**B**), tumor weight (**C**), tumor inhibition (**D**), DOX accumulation in tumors (**E**), and body weight (**F**) of A549 xenografts in nude mice after treatment with saline solution, NB, DOX, and NB+DOX. (**G**) Immunohistochemical and hematoxylin and eosin staining of tumor tissues from each group. Each value represents means ± SD.* i.v.*: intravenous administration; *p.o.*: oral administration, N: NB, D: DOX; Bars with different characters (a-d) are statistically different at *P* < 0.05. Statistical difference was assessed between groups (n = 8), which is represented as statistical difference of the final tumor volume in these treatment groups (as indicated by the cyan box). *P < 0.05, **P < 0.01, n = 8.

To evaluate the potential therapeutic efficacy of NB and DOX, we used a xenograft nude mice model assay. Both NB and DOX were inhibited tumor growth (**Figure 6A-C**). After treatment for 15 days, tumor volume decreased to 268.45 mm^3^ ± 17.01, and the tumor inhibition rate was 39.79%. However, tumor volume was significantly inhibited in the combined treatment with NB and DOX. The average tumor volume of the *p.o.* NB + *i.v.* DOX and *i.v.* NB + *i.v.* DOX groups was 205.61 mm^3^ ± 17.05 and 164.42 mm^3^ ± 25.03, respectively. The tumor inhibition rate was 53.95% and 63.16% in the *p.o.* NB + *i.v.* DOX and *i.v.* NB + *i.v.* DOX groups, respectively (**Figure 6D**). Additionally, we found that intravenous injection of NB and DOX had a more potent anti-tumor effect than did an oral administration schedule. This may be the result of higher NB bioavailability after intravenous administration 28, which promotes DOX accumulation in the tumor site and induces more significant anti-tumor effects. The stability evaluation of NB under different conditions (**Figure S13**) and DOX accumulation in tumor tissues also confirmed this result. For instance, the content of DOX was 1.89 (μg/g tumor tissue) ± 0.05 in DOX treatment groups and 3.34 (μg/g tumor tissue) ± 0.69 in *p.o.* NB + *i.v.* DOX groups. However, DOX concentration in the tumor tissues of* i.v.* NB + *i.v.* DOX groups was much higher with DOX content at 5.27 (μg/g tumor tissue) ± 0.41 (**Figure 6E**). And no significant body weight changes were found during the treatment (**Figure 6F**). Ki67 expression in tumor sections also verified these findings (**Figure 6G**). Moreover, no significant pathological changes in the major organs of heart, liver, spleen, lung, and kidney and TG, UA, ALB, TP, and LDH were observed ed between the combined treatments groups and control groups (**Figure S14**). Taken together, these results suggest that NB and DOX act synergistically to suppress tumor growth *in vivo*.

## Discussion

The search for phytochemicals with anticancer activities for application in chemotherapy and chemoprevention is an area of intense research. Most phytochemicals are used as adjuvants or enhancers due to weaken anticancer activities when used alone. The development of chemosensitizers with high efficiency, low toxicity, and complementary molecular mechanisms to decrease the dose-related adverse effects of chemotherapeutic agents is of great significance. We found that NB (dextrorotatory borneol ((+)-borneol)) was able to enhance the therapeutic efficiency of DOX in A549 cells. MS-SPRi results and MOE docking analysis suggest that TRPM8 may be the candidate target of NB as chemosensitizers of DOX for human A549 tumor treatment.

The cell membrane and membrane located transporters are greatly affected by the cellular uptake and bioavailability of drugs. Depletion of cholesterol, destruction of the structural integrity of membrane lipid raft, and inhibition of P-gp expression can facilitate the anticancer efficiency of therapeutic agents 41,42. NB is often used in combination with aspirin, verapamil, and antipyrine because of its potent penetration properties, which can promote the absorption of drugs in the transdermal and mucosal systems, and facilitate the blood-brain barrier permeability of drugs 43. Studies have speculated that affecting the fluidity of the plasma membrane phospholipid bilayer and inhibiting the tight junction protein expression may account for the transmembrane absorption of NB 16,17,44. In this study, we found that NB treatment greatly affects the biological function of P-gp, as indicated by increased rhodamine 123 red fluorescence intensity after NB treatment, suggesting that enhanced cellular DOX uptake may account for the P-gp dysfunction. The SPRi-MS results reveal that ABCB5 and MDR3 are captured by NB, which further supports this hypothesis. Previously, we found that NB could synergize with selenocystine, curcumin, and bisdemethoxycurcumin to induce significant anticancer activities 18-20. However, the exact mechanisms and the therapeutic target of NB remained elusive. Furthermore, we found that NB could not work with all cytotoxic cancer agents. For instance, NB could not enhance the anticancer efficiency of cisplatin, paclitaxel, and DOX against A375 melanoma cells. The IC_50_ of these agents against A375 cells was 3.41, 0.65, and 0.43 μM, for cisplatin, paclitaxel, and DOX, respectively. However, when combined with NB treatment the IC_50_ values were 3.42, 2.3, and 0.34 μM, for cisplatin, paclitaxel, and DOX, respectively (data not shown). These results suggest that the enhanced anticancer activities of NB may not be solely ascribed to enhanced cellular uptake of therapeutic agents.

In addition to ABCB5 and MDR3, we found that TRPA1 and TRPM8 scored well in the MS analysis. TRPA1 may not participate in the synergistic anticancer activities of NB and DOX (**Figure S15**), even though TRPA1 mediates resistance in chemotherapies 45. TRPM8 induces drug resistance through regulating the protein expression levels of P-gp, MRP-2, and LRP 46. TRPM8 expression is up-regulated in several common human cancers, including prostate, lung, and breast cancer. The relationship between TRPM8 overexpression and lung cancer invasive phenotypes has also been investigated 15. Additionally, TRPM8 is considered a cancer marker of lung cancer 35. Lung adenocarcinoma overexpresses TRPM8, and this expression is negatively correlated with survival rate in patients according to the TCGA and PROGgene database (**Figure S10**) 47,48, suggesting an important role for TRPM8 in lung cancer intervention. We found that TRPM8 is expressed in the plasma membrane and endoplasmic reticulum. We also found that NB was able to form a hydrogen bond with the side chain of Arg^841^, the critical residue for menthol activation of human TRPM8 38. Additionally, the NB hydrophobic bridge ring can interact interactions with hydrophobic amino acids Tyr^745^, Ile^845^, and Val^742^. Moreover, Tyr^745^ is the critical residue for menthol binding instead of cold sensing, and displays central roles in ligand-dependent gating in TRPM8 39. These results suggest that NB may activate TRPM8 through direct binding. Interestingly, we found that NB was able to dose-dependently trigger intracellular Ca^2+^ mobilization in A549 cells. This may be the result of NB passing through the cell membrane and binding TRPM8 in the endoplasmic reticulum; this could activate TRPM8 and trigger calcium ion efflux. Furthermore, TRPM8 knockdown suppressed the capacity of NB to trigger calcium mobilization (**Figure 5E**) as well as the killing potency of DOX in A549 cells sensitized by NB. Therefore, TRPM8 may be the candidate target of NB in sensitizing cells toward DOX and combating A549 tumor growth.

Evidence discloses that activation of Ca^2+^-mediated signaling pathway promotes cancer progression 49. However, excessive Ca^2+^ intracellular exhibits a critical role in regulating apoptosis through affecting the function of BAX and BAK 50. TRPM8 stimulation may result in calcium mobilization and *∆ψm* dissipation, promoting the release of cytochrome c and other contents which activate caspase-9 and caspase-3 to induce apoptosis. Herein, we found that NB could induce intracellular calcium accumulation in A549 cells in a dose-dependent manner. Moreover, TRPM8 knockdown restrains calcium accumulation, suggesting a pivotal role for TRPM8 in NB-triggered calcium mobilization. Additionally, NB was able to induce mitochondrial breakage, and up-regulate BAX and cleavage of Bid (tBid) expression and trigger mild levels of apoptosis (the proportion of apoptotic cells was 13%), which may be attributed to the calcium regulation induced by NB.

Triggering extrinsic and intrinsic apoptotic pathway are involved in the anticancer activities of DOX 51. The extrinsic and intrinsic apoptosis pathway activation is activated by caspase-8 and caspase-9 activation, respectively. The death receptor can be activated by binding with its specific ligand and result in activation of caspase-8 52. The activation of caspase-8 can induce tBid, which may translocate to the membrane of mitochondria and promote oligomerization of BAK and BAX and thus contribute to the activation of intrinsic apoptosis pathway. The integrity of the mitochondrial membrane is regulated by the balance of pro-survival proteins, including Bcl-2 and Bcl-xL, and pro-apoptosis proteins, including BAX and Bad. Upon apoptosis inducing signal transduction, loss of ∆*ψ*m will ultimately results in the destruction of mitochondrial structure 9. Additionally, mitochondria has also been confirmed to be an important target in chemotherapy 53. This study shows that NB combined with DOX dramatically activates caspase-8 and caspase-9, which suggests the activation of extrinsic and intrinsic apoptosis pathway. Additionally, the combined treatment of NB and DOX downregulates Bcl-2/Bax protein expression to induce mitochondrial dysfunction and activate the endogenous apoptotic pathway. This suggests that the mitochondria-mediated pathway plays an important role in NB sensitization of DOX.

ROS plays important regulatory roles in chemotherapy 54,55, and the mitochondria are potential source of ROS 56. Many studies have also disclosed the roles of ROS in the anticancer activities of DOX or DOX encapsulating particles 57,58. We found that both DOX and NB could induce intracellular ROS production, and significant ROS accumulation was observed following combined treatment with NB and DOX. This is consistent with the mitochondrial structural damage results, membrane potential dissipation, and up-regulation of pro-apoptotic protein expression induced in the combination treatment groups. Furthermore, NAC effectively inhibited this synergistic effect, indicating the importance of ROS in this synergism. Moreover, intracellular ROS production induction was significantly attenuated in the combined treatment group after TRPM8 knockdown. These results indicate that TRPM8 plays an important role in ROS overproduction-mediated apoptosis induced by NB and DOX.

The activation of p42/44MAPK (ERK1/2) and AKT signaling pathways are important features of tumor proliferation and invasion. Calcium signaling is involved in regulating the p44/p42-MAPK and PI3K-AKT-mTOR signaling pathways 59,60. When the intracellular calcium exceeds a certain threshold, it may inhibit the p44/p42-MAPK pathway and lead to crosstalk between other signaling pathways. Additionally, MAPK and AKT are the main oxidative stress sensitive signal transduction pathways. Consistent with reported studies, we found that DOX can activate p38 MAPK and JNK signals 61. We found that both NB and DOX could inhibit the expression of phosphate ERK1/2, but the combination of NB and DOX exhibited a synergistic inhibitory effect on the expression of phosphorylated AKT and ERK1/2. Additionally, NB combined with DOX induced more significant expression of phosphorylated p38MAPK and phosphorylated JNK. The underlying mechanism may be that NB stimulates the release of calcium ions through interacting with TRPM8, which results in the translocation of Ca^2+^ to mitochondria and induces mitochondrial damage leading to ROS production. ROS modulates MAPK, AKT signal transduction and enhances the therapeutic effects of DOX.

In response to DNA damage, downstream molecules including ATM and ATR can be recruited and activated, which activates p53 and ultimately induces Bax, PUMA, Noxa, and Bid expression. Strategies that target and activate p53 are important for sensitization treatment. DOX is a potent apoptosis-inducing chemotherapeutic agent that correlates with DNA damage, ROS generation, and p53 activation. We found that NB sensitized A549 cells to DOX-mediated killing through inducing DNA damage and activating the p53 signaling pathway. Furthermore, the synergism between NB and DOX is dependent on ROS generation, suggesting a critical role for ROS in this synergism.

We found that the proteins captured by NB, such as AR and RFA1, have relatively high MS scores and abundance on chips (PSMs). Additionally, AR and RFAI are closely related to the development of cancer, including lung, prostate, liver, and breast cancer 37. TCGA database data show that in human lung cancer tissue RFA1 is highly expressed (*P* = 1.09E-06), rendering it a therapeutic target for lung cancer treatment 62. AR is associated with the development of lung cancer, and AR abnormal expression often leads to poor prognosis and affects the survival of patients with lung cancer 37. Androgen stimulation can greatly affect gene expression profiles 63. Signal transduction crosstalk between AR and EGFR often leads to p38MAPK-dependent activation of mTOR and cyclinD1 expression in prostate cancer cells and in lung cancer cells 64. There is an ARE element in the TRPM8 gene promoter, suggesting that AR can directly bind to, and regulate, TRPM8 expression. Evidence indicates that although TRPM8 expression is regulated by AR, there is little correlation between the calcium release triggered by TRPM8 and AR expression levels. For example, menthol can also regulate the mobilization of calcium induced by TRPM8 in AR-insensitive PC-3 cells 13. In this study, we found that the A549 cell cycle distribution did not significantly change after treatment with NB, and no significant changes RFAI expression were observed (data are not shown), indicating that NB may not change the biological function of RFAI. Similarly, the killing effect of NB combined with DOX in A549 cells was not affected after AR knockdown (**Figure S12**). These results suggest that NB may not enhance the anti-tumor activity of DOX through RFA1 and AR inhibition.

This study has several limitations. Although tumor xenograft experiments have been widely accepted for the evaluation of chemotherapy agent anticancer activities, there are limitations to the system because of significant species and immune system differences between humans and nude mice. These limitations could mean that a low-grade malignant human tumor cell line may become malignant tumor in nude mice model or vice versa, which would dramatically affect the purported therapeutic effects of chemotherapy agents. Therefore, the orthotopic tumor model may be more suitable for further evaluation of the synergistic anticancer effects of NB and DOX. Syngeneic model is one kind of tumor model that inoculated with homologous tumor cell line into the immunized inbred mice (such as C57BL/6 or BALB/c strains). This model has the advantages of simple operation, low cost, repeatability, rapid and synchronous tumor growth, and it is easy to objectively judge the curative effect. But most of the inoculated tumors come from the same kind of cancer cells cultured* in vitro*, which lacks the microenvironment for the growth of human tumors. Overall, syngeneic model may also suitable for the screening of new antineoplastic drugs. Therefore, it may also suitable to employ a syngeneic model to examine the anticancer effects of NB and DOX. Additionally, NB was able to capture many proteins. Therefore, based on the findings of this study, we still could not conclude that TRPM8 is the only target of NB in the sensitization of DOX in A549 cells and more technologies are needed to confirm the target evaluation.

## Conclusion

**Figure 7:**
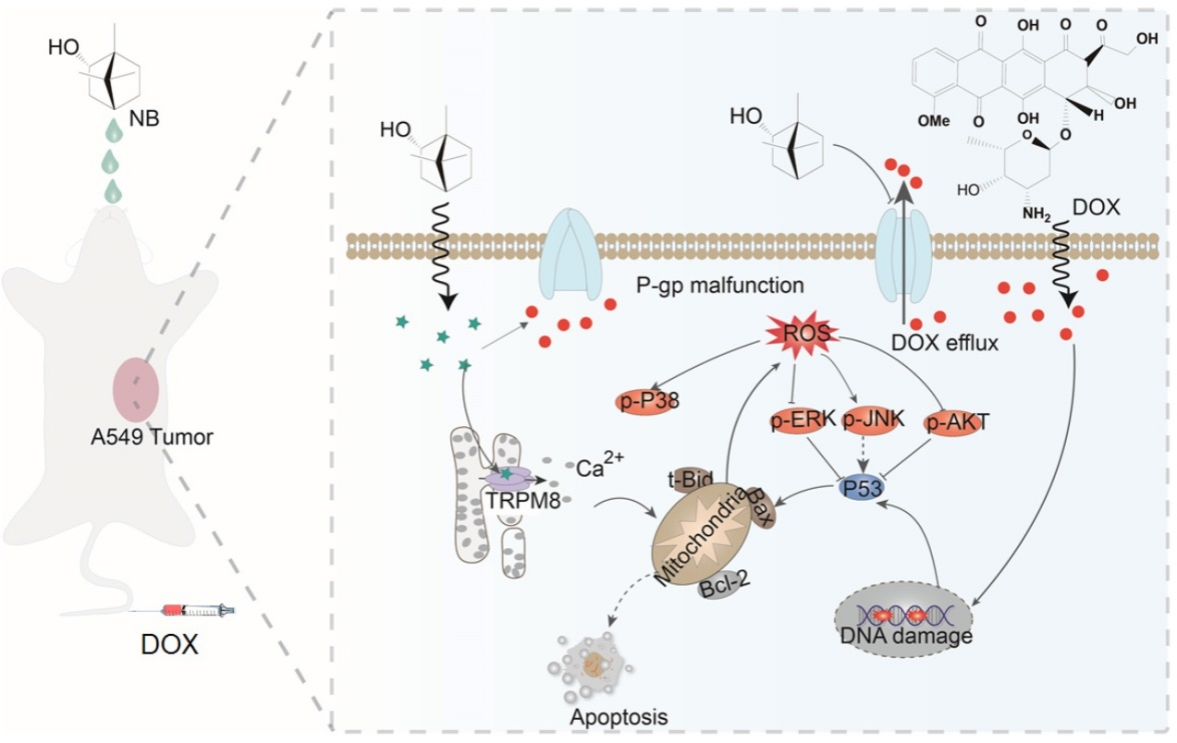
** The proposed signaling pathway induced by combination treatment with NB and DOX.** NB enhances DOX cellular accumulation and augments DNA damage. This activates the p53 pathway and results in activation of the mitochondria-mediated apoptosis pathway through downregulating the Bcl-2/Bax expression ratio. NB also triggers intracellular Ca^2+^ mobilization through interacting with TRPM8. Ca^2+^ overproduction may facilitate mitochondrial dysfunction and affect ROS generation. In turn, this can suppress activation of ERK1/2 and AKT, and facilitate p38MAPK and JNK phosphorylation to boost p53 activation and strengthen the effects on apoptosis.

Here, we have shown that NB augments the therapeutic effects of DOX *in vitro* and *in vivo* through TRPM8-regulated calcium mobilization. Therefore, we propose that NB suppresses the biological function of the multidrug resistance protein, P-gp, and enhances cellular uptake of DOX. This then augments DNA damage-mediated p53 activation, induces mitochondria dysfunction, and promotes apoptosis. Additionally, NB evokes Ca^2+^ signals and facilitates mitochondria dysfunction, which promotes ROS release and induces ERK1/2 and AKT inactivation and JNK and p38 MAPK activation to boost the p53-mediated apoptotic pathway (**Figure 7**). Together, these results suggest that NB may be developed as a potential chemosensitizer to improve the efficacy of DOX-based cancer therapy for the treatment of A549 lung cancer.

## Supplementary Material

Supplementary figures and tables.

## Abbreviations

NB: natural borneol ((+)-borneol)
SB: synthetic borneol
MS-SPRi: surface plasmon resonance (SPR) and LC-MS combined analysis
ROS: reactive oxygen species
TRPM8: transient receptor potential melastatin-8
NSCLC: Non-small cell lung cancer
∆*ψ*m: mitochondrial membrane potential
LD_50_: the median lethal dose
IC_50_: 50% inhibiting concentration
TCGA: The Cancer genome atlas
*i.v.*: intravenous administration
*p.o.*: oral administration
AR: androgen receptor
RFA1: replications factor A protein 1
