# Supplementary material for: TRPM8-regulated calcium mobilization plays a critical role in synergistic chemosensitization of Borneol on Doxorubicin
Source: Theranostics. 2020 Aug 13;10(22):10154–70. doi: 10.7150/thno.45861 (PMC7481425; doi:10.7150/thno.45861)
Supplement: Supplementary file 1 — Supplementary figures and tables. [file thnov10p10154s1.pdf]

Supporting Information

for

TRPM8-regulated calcium mobilization plays a critical role in synergistic chemosensitization of borneol on doxorubicin

Haoqiang Lai[1,2], Chang Liu[2,], Liyuan Hou[2], Wenwei Lin[2], Tianfeng Chen[2]* and An Hong[1]*

[1] Department of Cell Biology & Institute of Biomedicine, National Engineering Research Center of Genetic Medicine, Guangdong Provincial Key Laboratory of Bioengineering Medicine, College of Life Science and Technology, Jinan University, Guangzhou, 510632, China

[2] Department of Chemistry, Jinan University, Guangzhou, 510632, China

Correspondence: Corresponding authors. Jinan University, Guangzhou 510632, China. Tel.: + 86 020 85225962. E-mail addresses: tchentf@jnu.edu.cn (T. Chen); Tel: +086-020-85221983; Fax: +086-020-85221983; E-mail addresses: tha@jnu.edu.cn (Professor An Hong)

Materials and methods

Chemicals and reagents

PARP antibody (#9532S), Cleaved PARP (Asp214) antibody (Human Specific) (#9541), Caspase-9 antibody (#9502S), Caspase-8 antibody (#4790S), Caspase-3 antibody (#9662), Cleaved-Caspase-3 antibody (#9661S),) Bax antibody (#2774S), Bcl-2 antibody (#2872), Bid antibody (#2002S), Phospho-p44/42 MAPK (ERK1/2) (Thr202/Tyr204) antibody (#8544), p44/42 MAPK (ERK1/2) antibody (#9102), p38 MAPK antibody (#9212), Phospho-38 MAPK antibody (#9211), Phospho-AKT antibody (#4060S), AKT antibody (#4691S), Phospho-SAPK/JNK (Thr183/Tyr185) antibody (#9255), SAPK/JNK Antibody (#9252), Phospho-Histone H2A.X (Ser139) antibody (#2577), Phospho-ATM (Ser1981) antibody (#5883S), Phospho-ATR (Ser428) antibody (#2853), Phospho-p53 (Ser15) antibody (#9286), p53 antibody (#2527), Calnexin antibody (#2679), Androgen Receptor antibody (#5153S), Anti-rabbit

IgG, HRP-linked antibody (#7074) and Anti-mouse IgG, HRP-linked antibody (#7076) were purchased from Cell Signaling Technology (Beverly, MA). Anti-TRPM8 antibody was obtained from Abcam (ab85617) and Alomone labs (ACC-059). β-Actin antibody (A5441) was obtained from Sigma-Aldrich.

Results

Table S1. Acute oral toxicity evaluation of synthetic borneol and natural borneol ((+)- borneol).

Borneol	LD_{50} (mg/kg)	95 % confidence interval
Synthetic Borneol	3129 mg/kg	1750-5000 mg/kg
Natural Borneol ((+)-Borneol)	5000 mg/kg	2016-9810 mg/kg

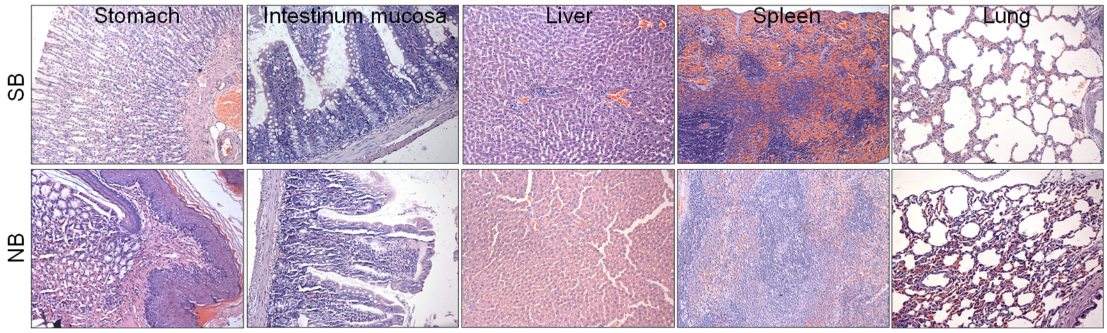

Figure S1. Histological analysis of stomach, intestinum mucosa, liver, spleen and lung of SD rats received with synthetic borneol and nature borneol ((+)-borneol) administration at 5 g/kg.

Table S2. Growth inhibition of the combination treatment of chemotherapeutic agents and NB against A549 cells.

Drugs	NB (μg/mL)			
IC_{50} (μM)	0	40	80	160
5-FU	>80	50.51±5.89	20.63±3.10	5.87±1.17
DOX	0.86±0.06	0.50±0.15	0.27±0.02	0.18±0.06
Paclitaxel	1.23±0.25	0.38±0.14	0.36±0.12	0.35±0.11
Cisplatin	3.90±0.86	4.05±1.95	3.52±0.53	1.87±0.54

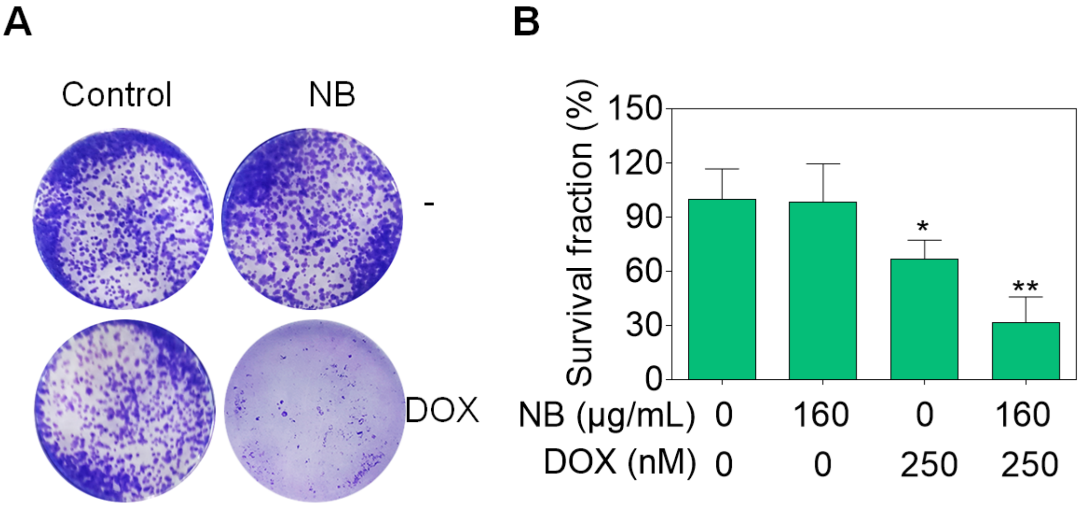

Figure S2. NB augments the suppression effect of DOX on the long term clonogenic assay. (A) Representative clonogenic images of A549 cells after the treatment of NB and DOX for 8 days. (B) Quantification of clones of A549 cells by manual counting. **P* < 0.05, ***P* < 0.001, when compared to the untreated control group (n=3).

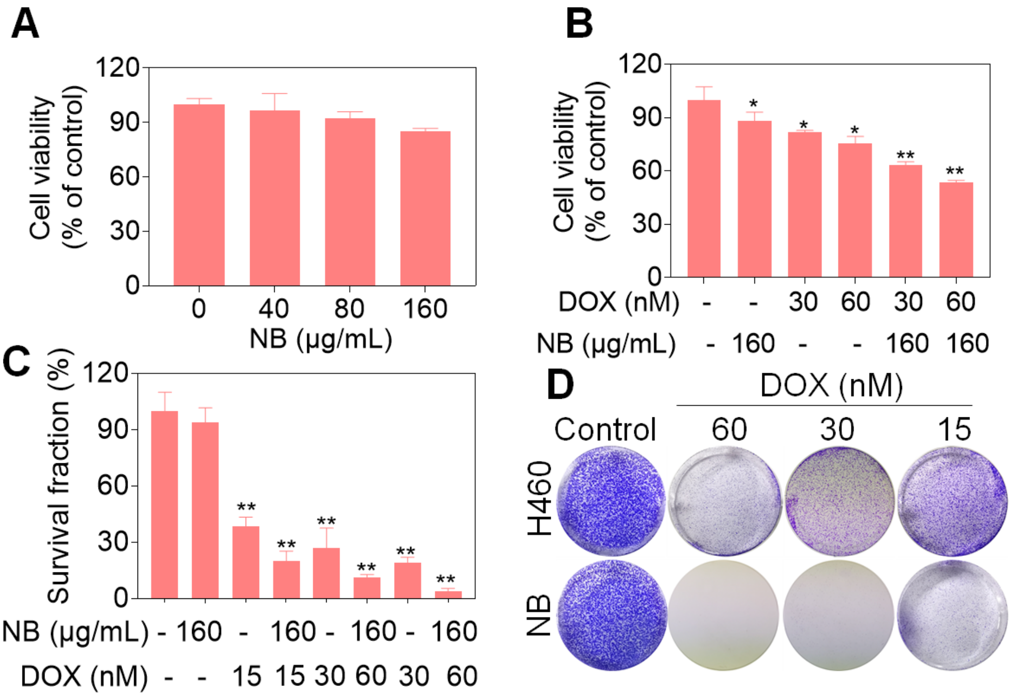

Figure S3. NB enhances the suppression effects of DOX against NCI-H460 cells. (A) Effects of NB (40, 80, and 160 μg/mL) on the survival of NCI-H460 cells. (B) NB synergizes with DOX to inhibit the proliferation of NCI-H460 cells. (C) NB pretreatment augments the inhibition effects of DOX against the clonogenic formation of NCI-H460 cells. (D) Representative images of clonogenic formation. **P* < 0.05, ***P* < 0.001, when compared to the untreated control group (n=3).

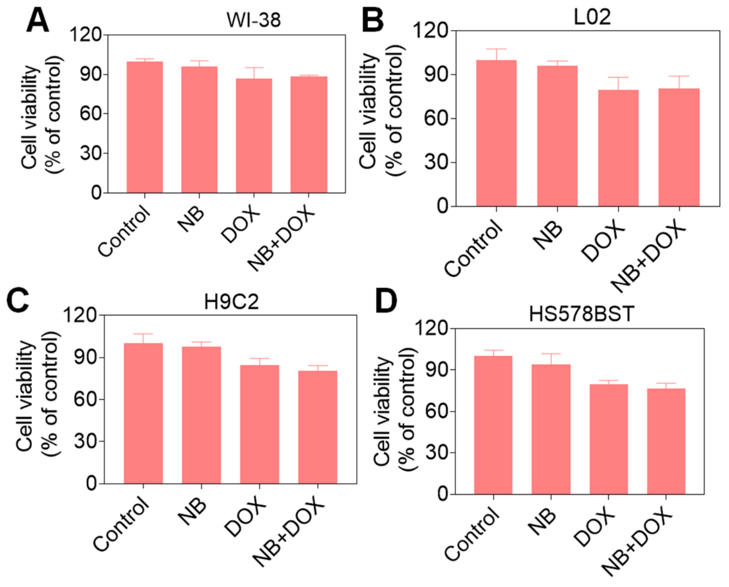

Figure S4. Cytotoxicity effects of the combination treatment of NB and DOX against normal cell lines. Cell growth suppression effects of the combined treatment of NB (160 μg/mL) and DOX (250 nM) in WI-38 cells (A), L02 cells (B), H9C2 cells (C) and HS578BST cells (D).

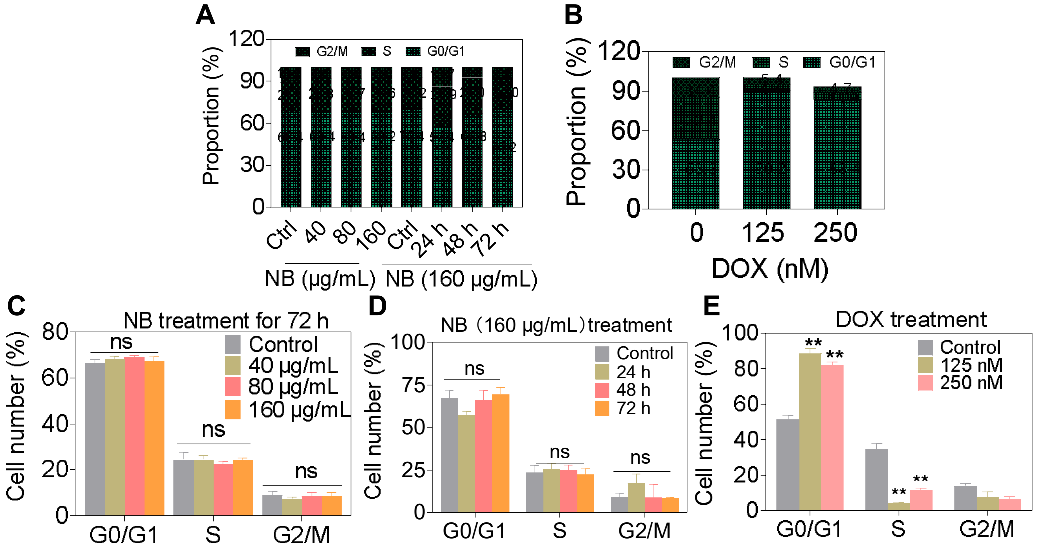

Figure S5. Cell cycle proportion quantification statistical analysis. Cells population in G0/G1, S and G2/M phase was quantified after the treatment of NB (A) and DOX (B). Statistical analysis of the cell cycle proportion after the treatment of NB (40, 80 and 160 μg/mL) for 72 h (C), NB (160 μg/mL) treatment for different times (24, 48 and 72 h) (D) and DOX (0.125 μM and 0.25 μM) for 72 h (E). ns refers to no significant differences. $^{**}P < 0.01$, (n=3).

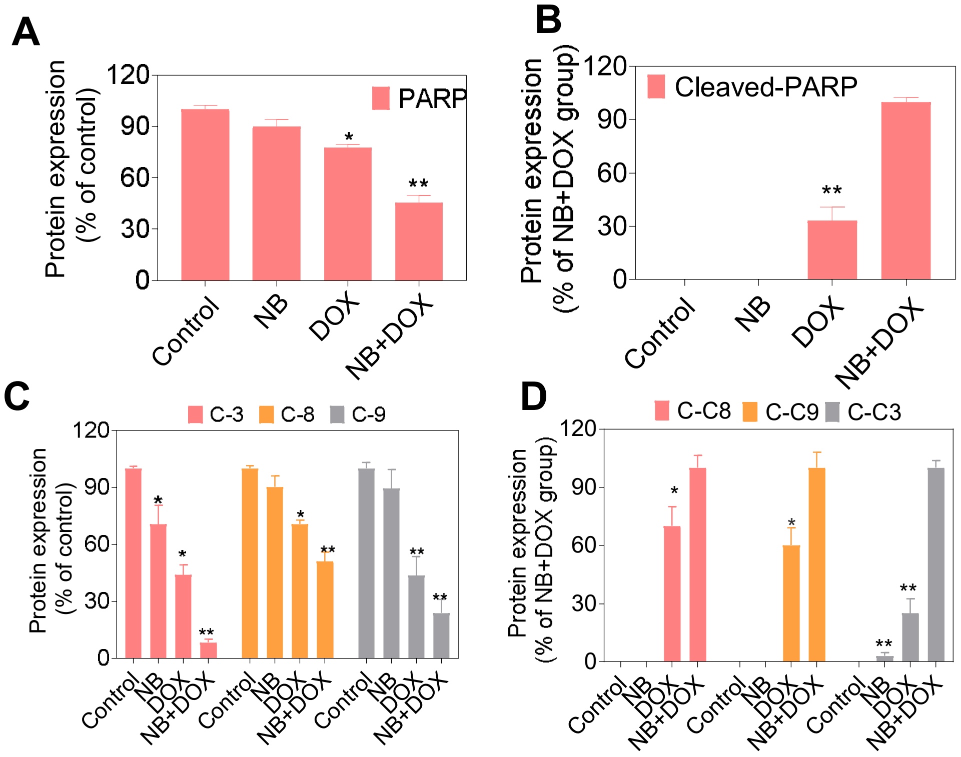

Figure S6. Statistical analysis of the protein expression after the combined treatment of NB and DOX. The protein expression of PARP (A), caspase-3/-8/-9 (C) are shown as the percentage of the control groups. (B) Cleaved PARP expression and cleaved caspase8, cleaved caspase-9 and cleaved-caspase3 (D) after the treatment of NB and DOX are shown as the percentage of the combined treatment groups of NB and DOX. **P* < 0.05, ***P* < 0.001, when compared to the control group or the combined treatment groups (n=3).

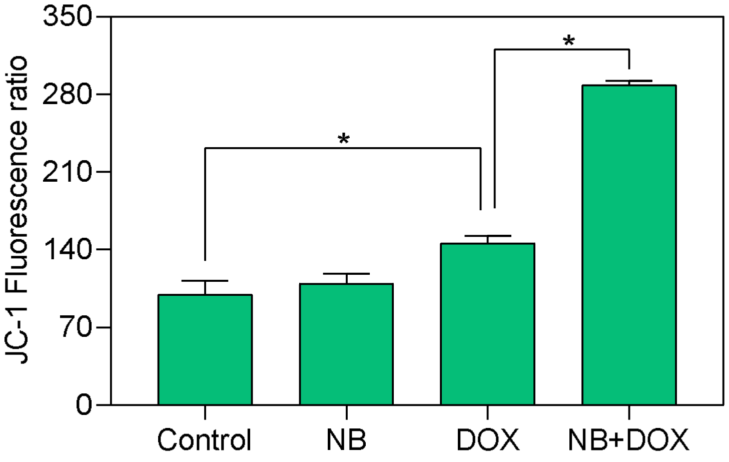

Figure S7. Effects of NB combined with DOX on mitochondria membrane depolarization by staining with JC-1 (2 μM). **P* < 0.05, n=3.

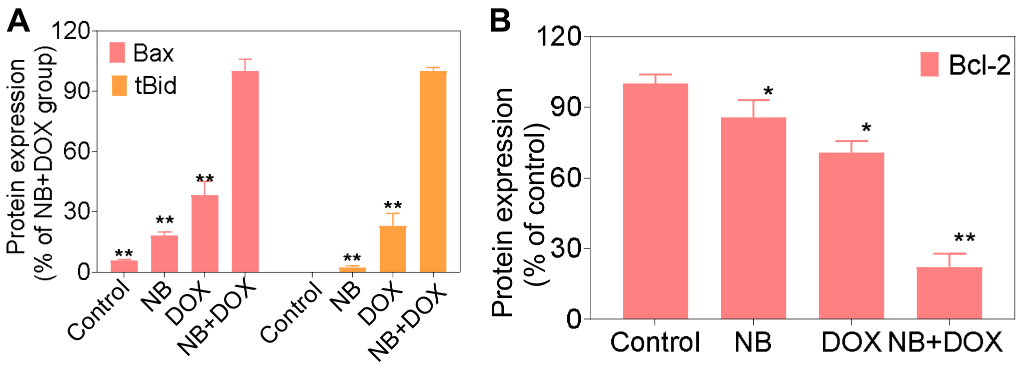

Figure S8. Statistical analysis of the protein expression level of Bax, tBid and Bcl-2. Protein expression of Bax and tBid are shown as the percentage of the combined treatment groups of NB and DOX (A). Bcl-2 protein expression level is shown as the percentage of the control groups. *$P < 0.05$, **$P < 0.001$, when compared to the control group or the combined treatment groups (n=3).

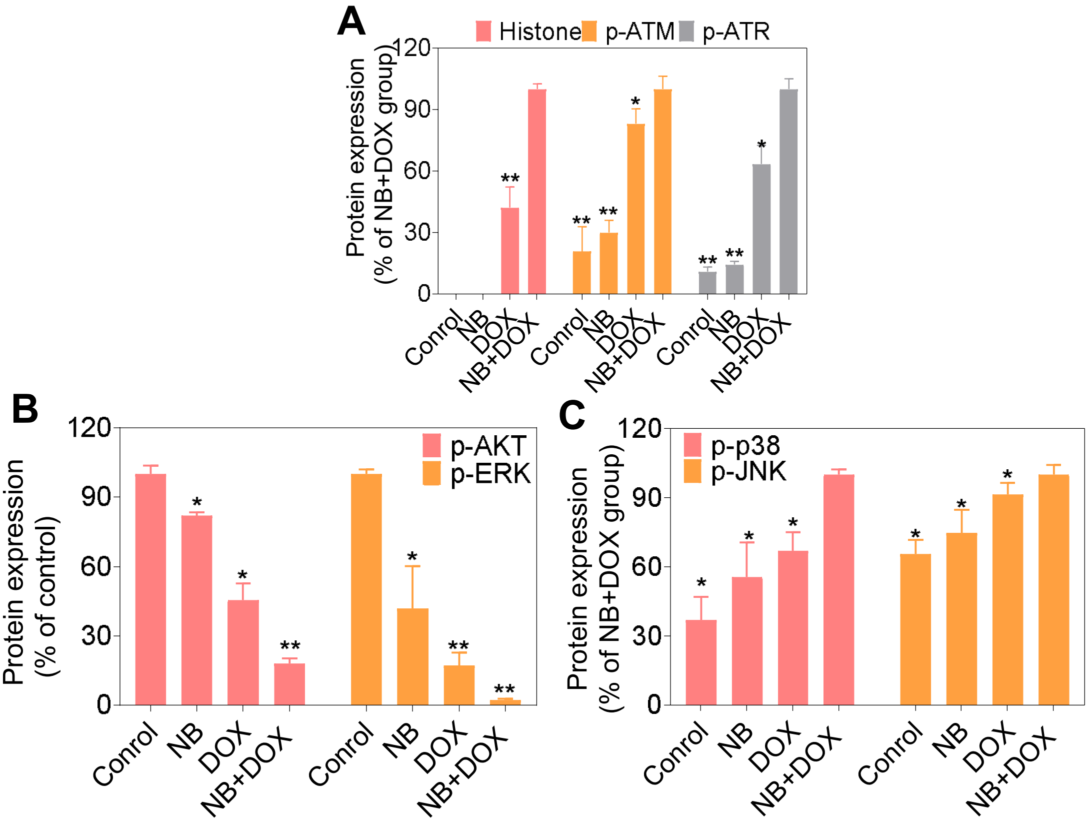

Figure S9. Statistical analysis of the protein expression level of histone, p-ATM, p-ATR (A), p-AKT, p-ERK (B), p-p38 and p-JNK (C) after the treatment of the combined treatment of NB and DOX. *$P <$ 0.05, **$P < 0.001$, when compared to the untreated control group or the combined treatment groups (n=3).

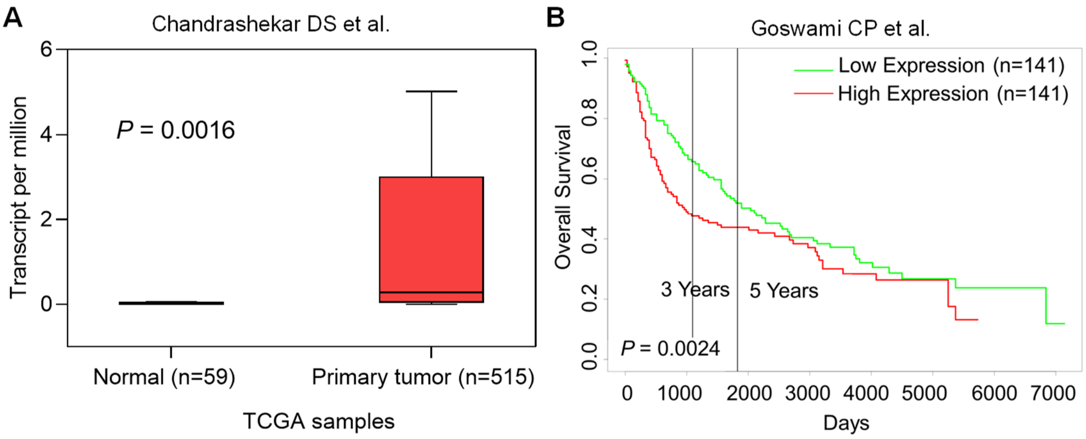

Figure S10. The relationship between the expression of TRPM8 and the patient survival of lung cancer. (A) Different expression level of TRPM8 between human normal lung tissue and lung cancer tissue. The data come from TCGA database [1] (http://ualcan.path.uab.edu/). (B) The relationship between the expression of TRPM8 in human lung cancer and the survival rate of patients. The data come from PROGene database [2] (http://genomics.jefferson.edu/proggene/).

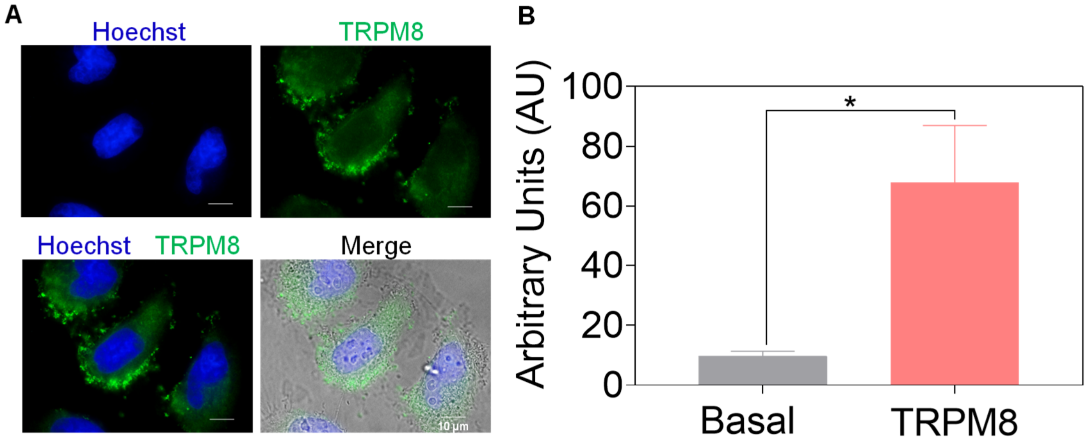

Figure S11. TRPM8 expression in A549 cells. (A) Immunofluorescence examination. (B) Statistical analysis of the expression of TRPM8 in A549 cells. The fluorescence intensity of TRPM8 of random five fields was quantified using Image-Pro Plus software (Media Cybernetics, USA). Basal refers to cells with no fluorescence. Statistical analysis was carried out using One-Way ANOVA in SPSS statistics 25 (SPSS statistics 25; SPSS, Inc. Chicago, IL). *$P < 0.05$, n=5.

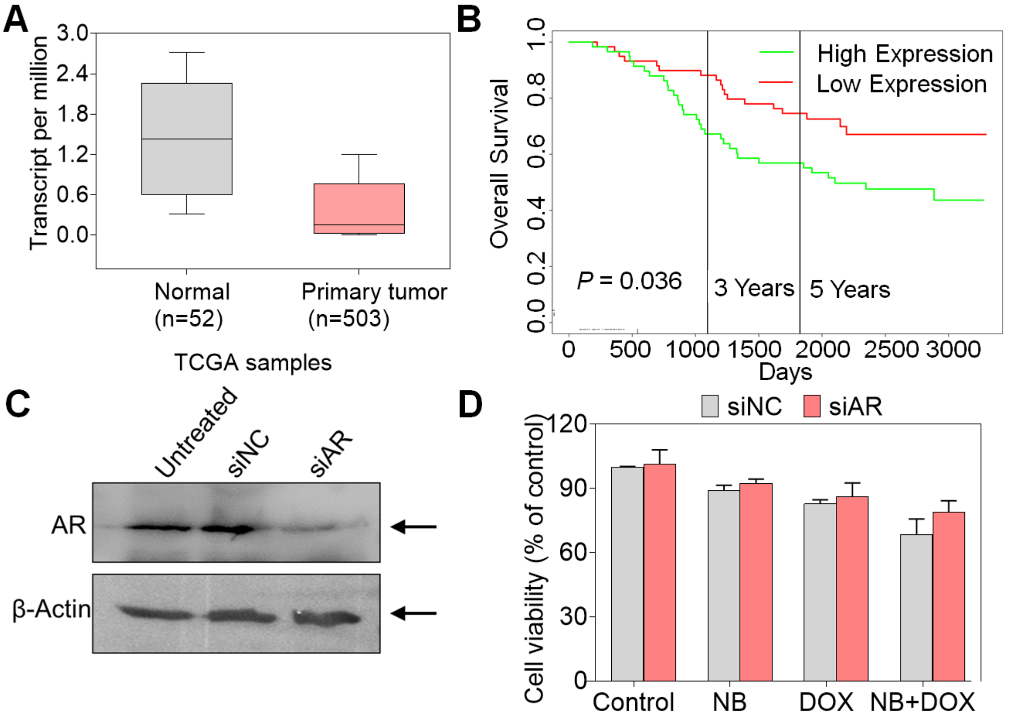

Figure S12. **The synergistic anticancer effects of NB and DOX is independent of androgen receptor (AR)-mediated pathway.** (A) The expression of AR in human lung normal tissue and lung cancer tissue. The data come from TCGA database [1]. (B) The relationship between the expression of AR in human lung cancer and the survival rate of patients. The data come from PROGene database [2]. (C) Evaluation of the expression of AR after cells transfected with AR siRNA. (D) Effects of the antitumor activities of NB combined with DOX after cells transfected with AR siRNA.

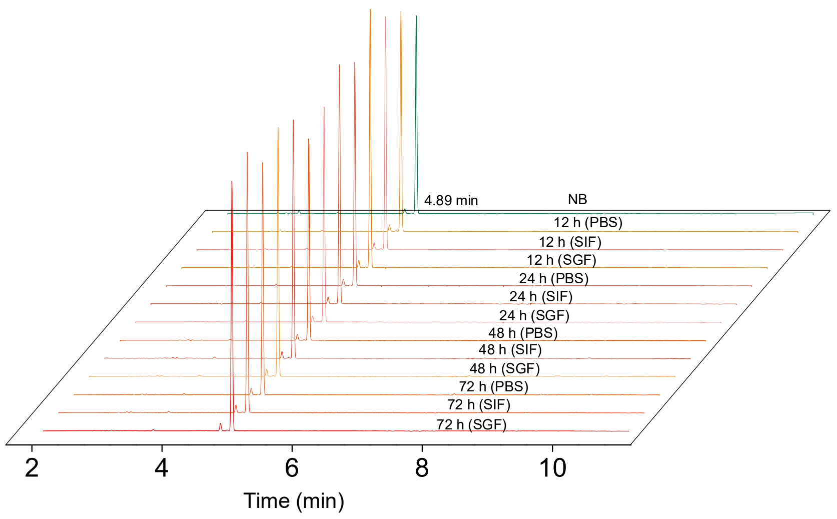

Figure S13. Stability analysis of NB under different condition using gas chromatography assay. SGF: simulated gastric fluid (pH=1.2-1.4), SIF: simulated intestinal fluid (pH=6.8). PBS (pH=7.4).

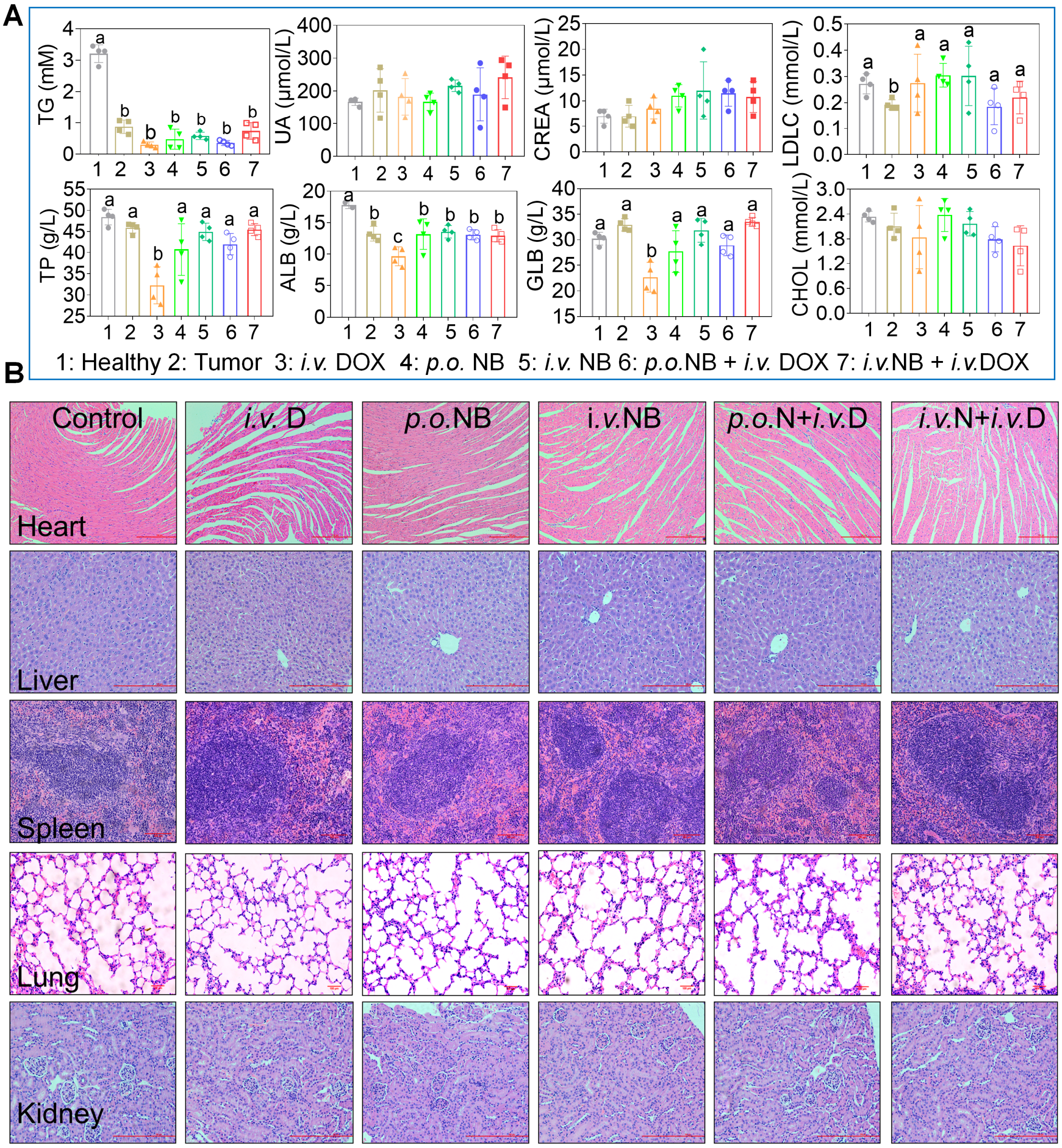

Figure S14. Histological and hematological examination. (A) Histological study of combined treatment with NB and DOX. (B) Hematological analysis of healthy, tumor-bearing, and NB and DOX combination treatment in nude mice. Each value represents means ± SD. *i.v.*: intravenous administration; *p.o.*: oral administration, N: NB, D: DOX; Bars with different characters (a–d) are statistically different at $P < 0.05$. Statistical difference was assessed between groups (n = 8), which is represented as statistical difference of the final tumor volume in these treatment groups (as indicated by the cyan box). *$P < 0.05$, **$P < 0.01$, n = 8.

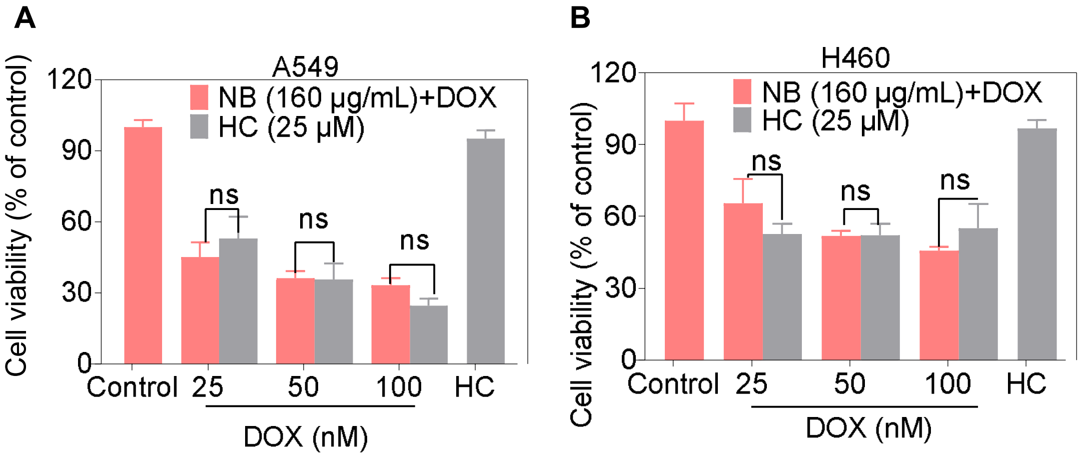

Figure S15. The synergistic anticancer effects of NB and DOX is independent of TRPA1-mediated pathway. The selective TRPA1 inhibitor HC-030031 pretreatment did not affect the cytotoxicity effects of the combined treatment of NB and DOX against A549 cells (A) and NCI-H460 cells (B).

References:

1. Chandrashekar DS, Bashel B, Balasubramanya SAH, Creighton CJ, Ponce-Rodriguez I, Chakravarthi BVSK, Varambally S: UALCAN: A Portal for Facilitating Tumor Subgroup Gene Expression and Survival Analyses. Neoplasia. 2017, 19:649-58.

2. Goswami CP, Nakshatri H: PROGgeneV2: enhancements on the existing database. BMC Cancer. 2014, 14. http://doi.10.1186/1471-2407-14-970.
